# Superenhancer activation of KLHDC8A drives glioma ciliation and hedgehog signaling

**DOI:** 10.1172/JCI163592

**Published:** 2023-01-17

**Authors:** Derrick Lee, Ryan C. Gimple, Xujia Wu, Briana C. Prager, Zhixin Qiu, Qiulian Wu, Vikas Daggubati, Aruljothi Mariappan, Jay Gopalakrishnan, Matthew R. Sarkisian, David R. Raleigh, Jeremy N. Rich

**Affiliations:** 1 UPMC Hillman Cancer Center, Pittsburgh, Pennsylvania, USA.; 2 Department of Medicine, University of Pittsburgh, Pittsburgh, Pennsylvania, USA.; 3 Division of Regenerative Medicine, Department of Medicine, UCSD, La Jolla, California, USA.; 4 Department of Pathology, Case Western Reserve University, Cleveland, Ohio, USA.; 5 Cleveland Clinic Lerner College of Medicine, Cleveland, Ohio, USA.; 6 Department of Radiation Oncology and; 7 Department of Neurological Surgery, UCSF, San Francisco, California, USA.; 8 Institute of Human Genetics, University Hospital Düsseldorf, Heinrich-Heine-Universität, Düsseldorf, Germany.; 9 Department of Neuroscience, McKnight Brain Institute and; 10 Preston A. Wells, Jr. Center for Brain Tumor Therapy, University of Florida, Gainesville, Florida, USA.; 11 Department of Neurology, University of Pittsburgh, Pittsburgh, Pennsylvania, USA.

**Keywords:** Oncology, Stem cells, Cancer

## Abstract

Glioblastoma ranks among the most aggressive and lethal of all human cancers. Self-renewing, highly tumorigenic glioblastoma stem cells (GSCs) contribute to therapeutic resistance and maintain cellular heterogeneity. Here, we interrogated superenhancer landscapes of primary glioblastoma specimens and patient-derived GSCs, revealing a kelch domain–containing gene, specifically Kelch domain containing 8A (KLHDC8A) with a previously unknown function as an epigenetically driven oncogene. Targeting KLHDC8A decreased GSC proliferation and self-renewal, induced apoptosis, and impaired in vivo tumor growth. Transcription factor control circuitry analyses revealed that the master transcriptional regulator SOX2 stimulated KLHDC8A expression. Mechanistically, KLHDC8A bound chaperonin-containing TCP1 (CCT) to promote the assembly of primary cilia to activate hedgehog signaling. KLHDC8A expression correlated with Aurora B/C Kinase inhibitor activity, which induced primary cilia and hedgehog signaling. Combinatorial targeting of Aurora B/C kinase and hedgehog displayed augmented benefit against GSC proliferation. Collectively, superenhancer-based discovery revealed KLHDC8A as what we believe to be a novel molecular target of cancer stem cells that promotes ciliogenesis to activate the hedgehog pathway, offering insights into therapeutic vulnerabilities for glioblastoma treatment.

## Graphical abstract

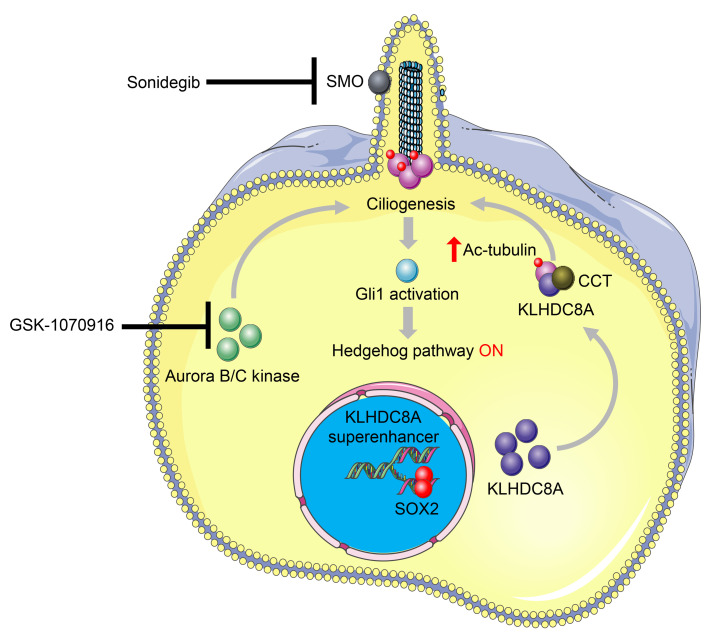

## Introduction

Glioblastoma is the most prevalent primary intrinsic brain tumor in adults, with a median survival of 12–5 months (1, 2). Standard-of-care treatment includes maximal surgical resection followed by chemoradiation with the oral methylator temozolomide and adjuvant temozolomide, which marginally improves patient survival (3). While glioblastoma has undergone extensive molecular characterization and classification into subtypes based on transcriptional profiles (4–6), translation of this knowledge to clinical practice is limited. Self-renewing, highly tumorigenic, and stem-like cancer cells, called glioblastoma stem cells (GSCs), contribute to therapeutic resistance and poor prognosis (7, 8). While the cell-of-origin and universal-identification markers specific for GSCs remain controversial, GSCs promote tumor angiogenesis, brain invasion, and immune evasion (9–11), highlighting the potential benefit in targeting GSCs.

Glioblastomas and other cancers have traditionally been viewed as a set of diseases that are driven by the accumulation of genetic aberrations. However, treatments focused on genetic drivers in glioblastoma have shown limited efficacy, suggesting that other therapeutic paradigms for targeting glioblastoma should be considered. More recently, an increasing body of evidence has emerged that epigenetic abnormalities, in concert with genetic alterations, drive cancer initiation and progression (12). Altered expression of epigenetic and chromatin regulators are linked to malignant phenotypes of glioblastoma (13). Superenhancers are clusters of putative enhancers in close proximity, with strong enrichment for the binding of master transcription factors and mediator coactivators, which drive high expression of genes that define cell state and control cell identity (14). We previously demonstrated that targeting ependymoma superenhancer-associated genes impaired the proliferation of patient-derived neurospheres and xenografts (15), suggesting that interrogation of superenhancers and their associated genes can provide insights into drug discovery and the mechanisms of disease pathogenesis.

Extracellular signaling is a crucial determinant of cancer cell proliferation, migration, and invasion. Signals derived from the tumor microenvironment are critical for cancer stem cell maintenance (16). Core stem cell pathways, such as WNT, NOTCH, and sonic hedgehog, promote stemness and inhibit apoptosis of cancer stem cells (17). The hedgehog pathway, which is mediated by primary cilia in a context-dependent manner, is commonly dysregulated in medulloblastoma and gliomas (18). The inhibitors of the hedgehog pathway have demonstrated clinical efficacy in the treatment of basal cell carcinoma and are under active investigation for other cancer types (19–21). However, resistance to hedgehog inhibitors has been reported (22, 23). In addition to genetic mutations in Patched 1 (PTCH1), Smoothened (SMO), or other hedgehog signaling components, which drive the constitutive activation of hedgehog pathway (24–26), epigenetic dysregulation also leads to aberrant hedgehog activation, and inhibition of epigenetic regulatory protein BRD4 downregulates hedgehog pathway genes and inhibits the growth of hedgehog-driven tumors resistant to Smoothened antagonists (27). Considering the functional importance of superenhancers and core stem cell pathways, we hypothesized that interrogation of glioblastoma-specific superenhancers and their associated genes by utilizing superenhancer profiling would uncover GSC biology and reveal critical dependencies in glioblastomas.

## Results

### Identification of epigenetically upregulated genes in GSCs.

**Figure 1:**
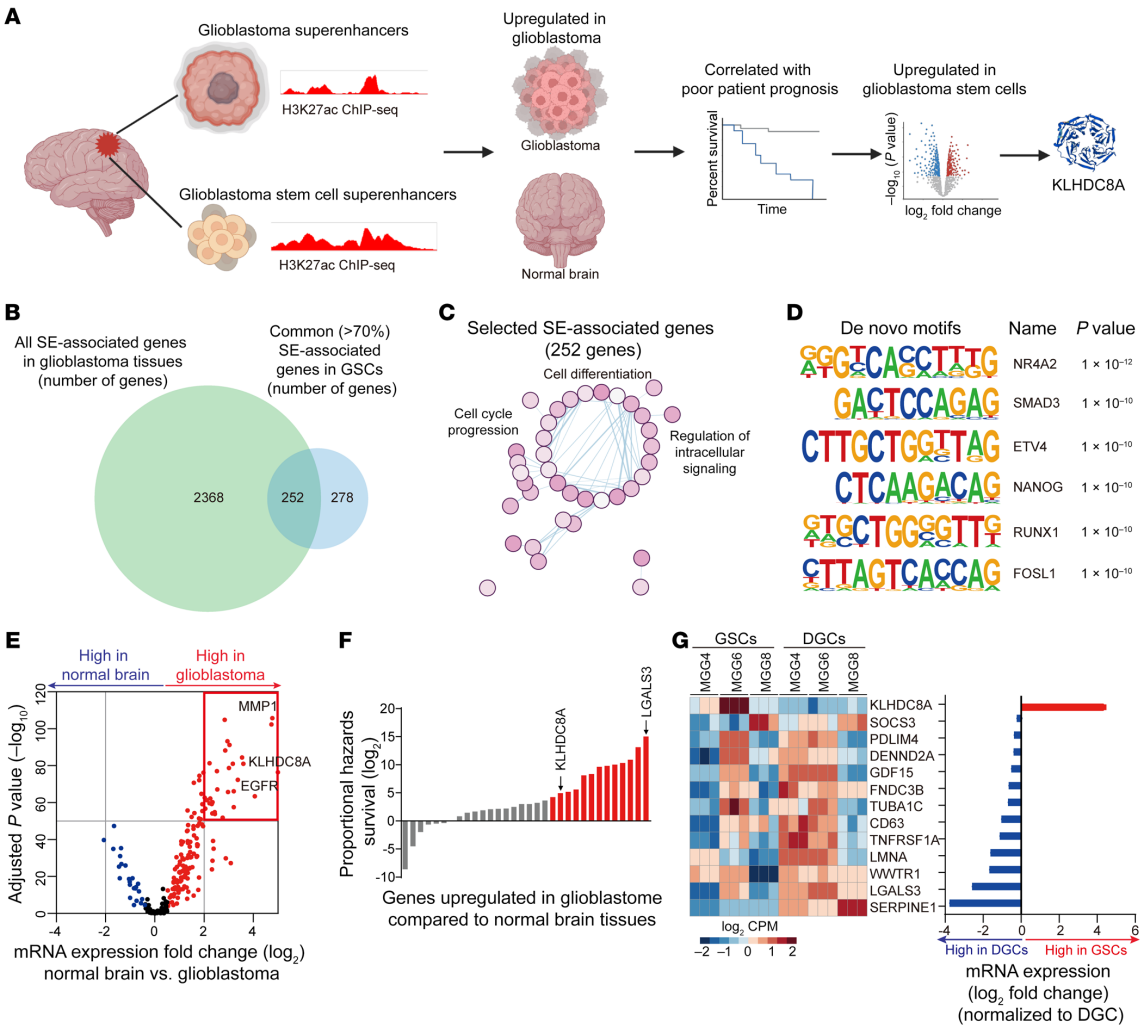
Superenhancer screen identified a potential GSC vulnerability. (**A**) Diagram depicting the superenhancer screen and target prioritization approach. (**B**) Venn diagram showing the intersection between all superenhancer-associated genes in 11 glioblastoma tissues and common (>70%) superenhancer-associated in 37 GSCs. (**C**) GSEA of Hallmark, curated, and GO pathways enriched for gene sets correlated with the H3K27ac signal intensity of GSC superenhancer-associated genes. (**D**) De novo HOMER motif analysis of 252 selected GSC superenhancers, as described in **B**. (**E**) Volcano plot showing the mRNA expression of selected superenhancer-associated genes in TCGA HG-U133A data set. Red dots indicate upregulated genes, while blue dots indicate downregulated genes in glioblastoma tissues compared with normal brain tissues. (**F**) Box plot showing the proportional hazards survival of 32 upregulated superenhancer-associated genes, as described in **E**. Red bars indicate the genes correlated with high proportional hazards survival at a log_2_ value greater than 4. (**G**) Heatmap and box plot showing the mRNA expression of 13 selected superenhancer-associated genes, as described in **F**, in 3 paired GSC and DGC models analyzed by R package Limma.

To identify GSC-specific epigenetic vulnerabilities, we performed unbiased *in silico* screening to identify superenhancer-associated genes specifically present in glioblastoma surgical specimens and patient-derived GSCs (Figure 1A). We profiled 11 glioblastoma surgical resection specimens for superenhancer loci via histone 3 lysine 27 acetylation (H3K27ac) chromatin immunoprecipitation followed by sequencing (ChIP-Seq) data sets (28, 29). To identify glioblastoma superenhancer-associated genes, we prioritized superenhancers present in all 11 glioblastoma tissues, yielding 2,620 genes regulated by glioblastoma superenhancers. Superenhancer-associated genes across 11 glioblastoma tissues included EGFR, POUF3, SOX2, and AVIL, which were each previously shown to contribute to glioblastoma tumorigenesis (Supplemental Figure 1A; supplemental material available online with this article; https://doi.org/10.1172/JCI163592DS1). Next, we interrogated GSC H3K27ac ChIP-Seq data sets (30) and identified the superenhancer loci and genes that are shared by more than 70% of GSCs; we hypothesized that these may represent key factors in control of GSC identity or tumorigenesis. We focused on the superenhancer-associated genes that were shared by glioblastoma tissues and GSCs to identify stem-specific features with in vivo relevance. There were 252 superenhancer-associated genes (Figure 1B) enriched for pathways involved in neural development, cell motility, cell cycle, and structure morphogenesis (Figure 1C and Supplemental Figure 1B). The selected GSC superenhancers were enriched for transcriptional motifs, including NR4A2, SMAD3, and ETV4, which have been previously reported to promote glioblastoma malignancy (31–33) (Figure 1D). The majority of superenhancers were located in the promoters and distal intergenic and intronic regions (Supplemental Figure 1C). Higher expression of superenhancer-associated genes informed poor prognosis of glioblastoma patients in The Cancer Genome Atlas (TCGA) and Chinese Glioma Genome Atlas (CGGA) data sets (Supplemental Figure 1D).

To prioritize among the 252 superenhancer-associated genes for further investigation, we took a 3-stage approach where (a) genes with elevated expression in glioblastoma tissues from TCGA data sets were compared with normal brain specimens from Genotype-Tissue Expression (GTex) data sets (Figure 1E); (b) genes for which high expression was associated with poor patient prognosis (Figure 1F); and (c) mRNA expression of 13 genes meeting these criteria listed in (a) and (b) were compared across 3 patient-derived GSCs and matched serum–differentiated glioma cells (serum-DGCs). Among the candidate genes, Kelch Domain Containing 8A (KLHDC8A) was the only gene displaying elevated expression levels in GSCs compared with DGCs (Figure 1G). Collectively, this superenhancer-identification approach strongly indicated KLHDC8A as a lead candidate for further investigation.

### KLHDC8A promoted GSC growth and maintenance.

**Figure 2:**
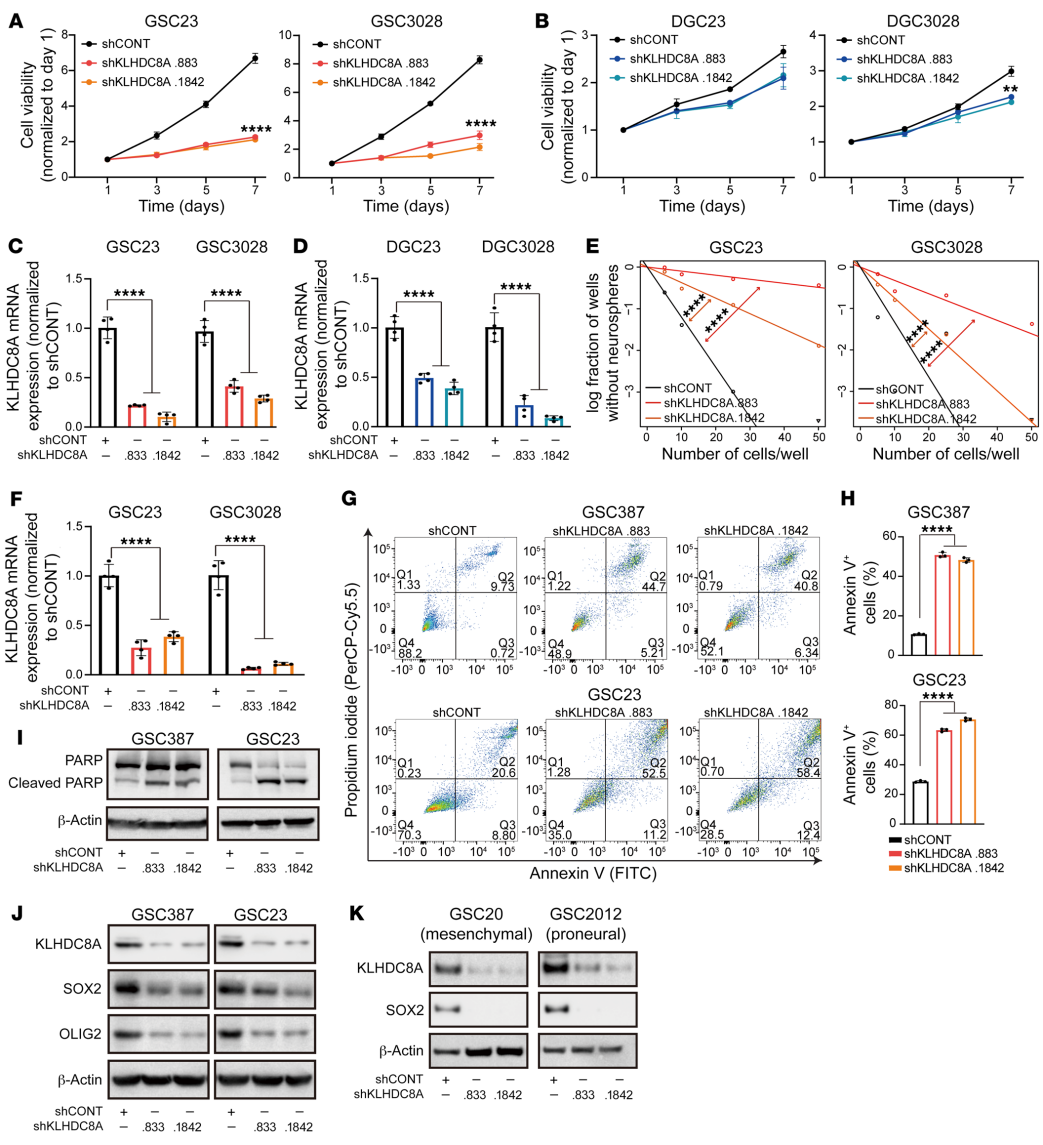
KLHDC8A is necessary for GSC maintenance. (**A** and **B**) Cell viability was measured by CellTiter-Glo assay in paired GSC23, GSC3028, and differentiated counterparts (DGC23 and DGC3028) over a 6-day time course following KLHDC8A knockdown. *n* = 4. Quantitative data from 4 technical replicates are shown as mean ± SD. Statistical analysis was performed using 2-way ANOVA with Dunnett’s multiple comparisons. (**C** and **D**) The knockdown efficiency of KLHDC8A was measured by qPCR in GSCs (**C**) and DGCs (**D**). *n* = 4. Quantitative data from 4 independent experiments are shown as mean ± SD. Statistical analysis was performed using 1-way ANOVA with Tukey’s multiple comparisons. (**E**) In vitro ELDA in GSC23 and GSC3028 following knockdown of KLHDC8A. 24 wells were quantified for each condition. Statistical analysis was performed using χ^2^ test for pairwise differences. (**F**) The knockdown efficiency of KLHDC8A was measured by qPCR in GSC3028 and GSC23. *n* = 4. Quantitative data from 4 independent experiments are shown as mean ± SD. Statistical analysis was performed using 1-way ANOVA with Tukey’s multiple comparisons. (**G**) Immunoblot showing protein levels of PARP and cleaved PARP in GSC387 and GSC23 following KLHDC8A knockdown. β-Actin was used as the loading control. (**H**) Annexin V staining of GSC23 and GSC387 was performed following knockdown of KLHDC8A. (**I**) Quantification of Annexin V staining in GSC387 and GSC23. *n* = 3. Quantitative data from 3 technical replicates are shown as mean ± SD. Statistical analysis was performed using 1-way ANOVA with Tukey’s multiple comparisons. (**J**) Protein levels of OLIG2 and SOX2 following KLHDC8A knockdown were measured by immunoblot. β-Actin was used as the loading control. (**K**) Protein levels of SOX2 following KLHDC8A knockdown in mesenchymal and proneural subtypes of GSCs were measured by immunoblot. β-Actin was used as the loading control. **P < 0.01, ****P < 0.0001.

To interrogate the functional importance of KLHDC8A in GSCs, KLHDC8A was targeted by shRNA-mediated knockdown in patient-derived GSCs and matched DGCs. We used 2 nonoverlapping shRNAs targeting KLHDC8A and compared them with a nontargeting control shRNA sequence (shCONT) that does not match any sequence in the mammalian genome. Inhibition of KLHDC8A expression impaired proliferation in GSCs, whereas targeting KLHDC8A marginally reduced the proliferation of DGCs (Figure 2, A–D). To rule out the possibility of shRNA off-target effects, exogenous KLHDC8A not targeted by the shRNA was expressed in KLHDC8A-knockdown GSCs. This overexpression of KLHDC8A rescued the GSC proliferation (Supplemental Figure 2, A and B). Extreme limiting dilution assay (ELDA) is a surrogate of self-renewal capacity, which is one of the defining characteristics of a stem cell. Upon downregulation of KLHDC8A, stem cell frequency and self-renewal capacity were diminished in 2 patient-derived GSCs (Figure 2, E and F). GSCs transduced with KLHDC8A shRNAs showed increased apoptotic cell death, as measured by Annexin V apoptotic assay and cleavage of poly(ADP-ribose)polymerase-1 (PARP1) (Figure 2, G–I). To determine the specific role of KLHDC8A in glioblastoma, we interrogated the functional importance of KLHDC8A in several nonneoplastic neural cells, including neural stem or progenitor cells (NSCs or NPCs) and nonmalignant neural cells (NMs) derived from epilepsy surgical-resection specimens. Depletion of KLHDC8A impaired the proliferation of NSCs but had minimal effect on the proliferation of nonmalignant brain cultures, indicating a potential role of KLHDC8A in regulating stemness of GSCs and NSCs (Supplemental Figure 2, C and D). As expected, KLHDC8A knockdown decreased the expression of GSC markers, OLIG2 and SOX2, in all 3 subtypes of GSCs (Figure 2, J and K). To understand the role of KLHDC8A across cell types and tissues, we interrogated The Cancer Dependency Map (Depmap) portal (www.depmap.org), which contains whole-genome CRISPR-knockout screen data across 558 cell lines. KLHDC8A was not a pan-essential gene in a panel of cancer types (Supplemental Figure 2E), which underscores the potential value of targeting KLHDC8A in glioblastoma. In sum, KLHDC8A plays a critical role in GSC proliferation, maintenance, and survival.

### Transcriptional regulation of KLHDC8A in GSCs.

**Figure 3:**
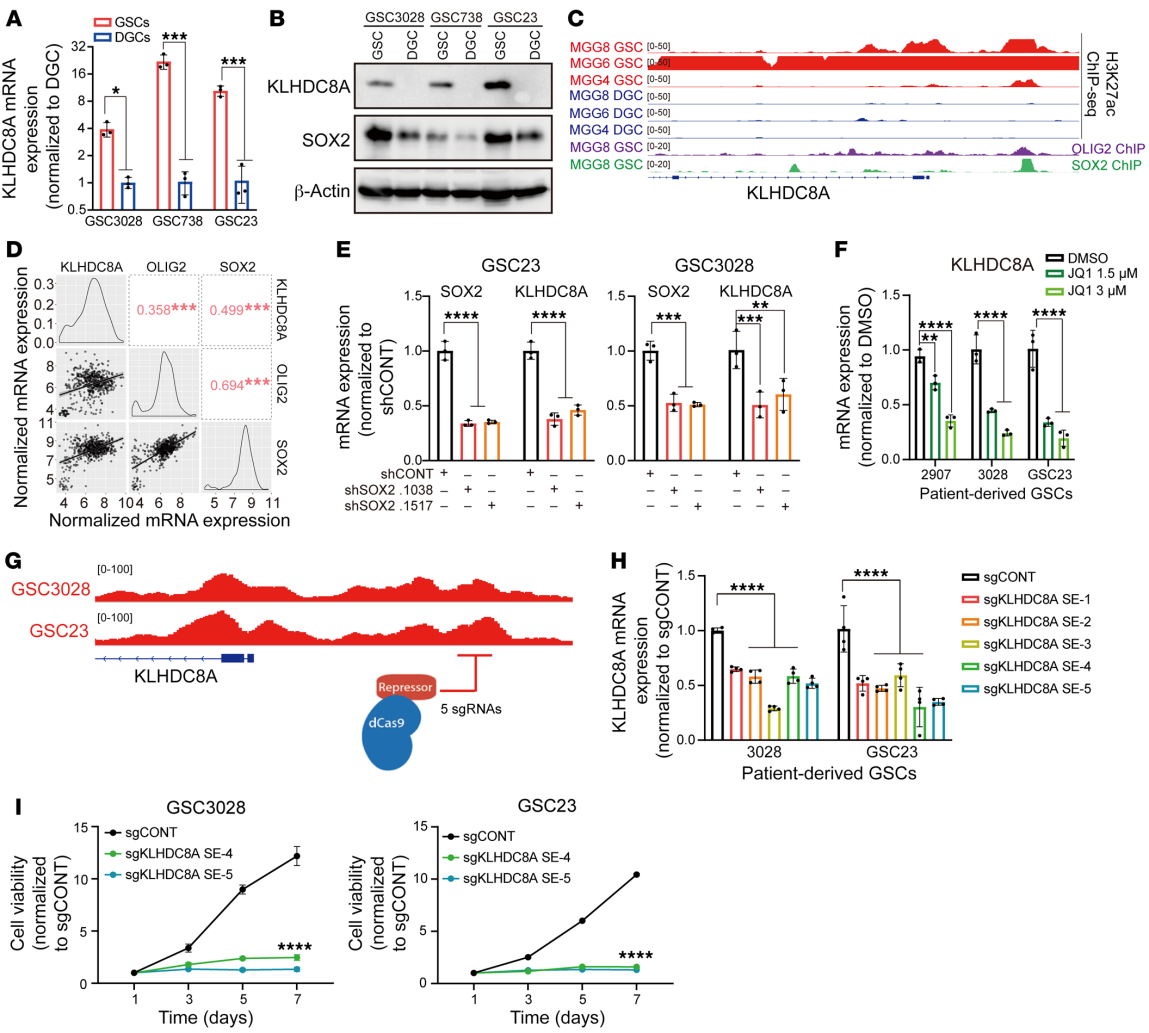
KLHDC8A expression is driven by SOX2 and a GSC superenhancer in GSCs. (**A**) KLHDC8A mRNA expression was measured in 3 matched pairs of GSCs and DGCs by qPCR analysis. *n* = 3. Quantitative data from 3 independent experiments are shown as mean ± SD. Statistical analysis was performed using Student’s t-test with the Holm-Šidák multiple test correction. (**B**) Protein levels of KLHDC8A were measured by immunoblot following KLHDC8A knockdown. SOX2 was used as the stemness marker. β-Actin was used as the loading control. (**C**) H3K27ac signals at the KLHDC8A superenhancer region in 3 pairs of GSCs and DGCs (MGG4, MGG6, and MGG8). SOX2 and OLIG2 ChIP-Seq signals are shown at the superenhancer region of MGG8. (**D**) Correlation of mRNA expression between KLHDC8A, OLIG2, and SOX2 in the TCGA HG-U133A glioblastoma data set. Numbers indicated the R value of Spearman correlation. (**E**) qPCR analysis of mRNA expression of SOX2 and KLHDC8A upon knockdown of SOX2. *n* = 3. Quantitative data from 3 independent experiments are shown as mean ± SD. Statistical analysis was performed using 2-way ANOVA with Dunnett’s multiple test correction. (**F**) qPCR analysis of mRNA expression of KLHDC8A following treatment with 2 concentrations of JQ1 (1.5 and 3 μM) for 24 hours. *n* = 3. Quantitative data from 3 independent experiments are shown as mean ± SD. Statistical analysis was performed using 2-way ANOVA with Dunnett’s multiple test correction. (**G**) Schematic displaying targeting of the KLHDC8A superenhancer region using dCas9-KRAB system with 5 nonoverlapping sgRNA targeting critical KLHDC8A superenhancer locus. (**H**) The mRNA expression of KLHDC8A in GSC23 and GSC3028 was measured by quantitative PCR. *n* = 4. Quantitative data from 4 independent experiments are shown as mean ± SD. Statistical analysis was performed using 2-way ANOVA with Dunnett’s multiple comparison. (**I**) Proliferation of GSCs measured by CellTiter-Glo assay in GSC23 and GSC3028 overexpressing dCas9-KRAB and 5 sgRNAs over a 6-day time course. *n* = 4. Quantitative data from 4 technical replicates are shown as mean ± SD. Statistical analysis was performed using 2-way ANOVA with Dunnett’s multiple test correction. *P < 0.05, **P <0.01, ***P < 0.001, ****P < 0.0001.

To define the epigenetic regulation of KLHDC8A, we interrogated the chromatin landscape of KLHDC8A in a cohort of patient-derived GSCs, 3 matched DGCs, and 3 NMs, which revealed strong enrichment of active chromatin regions in close proximity to KLHDC8A gene promoter region in GSCs (Supplemental Figure 2F). In accordance with strong H3K27ac signals within the superenhancer region, GSCs displayed elevated mRNA and protein expression of KLHDC8A compared with DGCs (Figure 3, A and B). Differentiation of GSCs was validated by downregulation of GSC-related transcription factors SOX2 and OLIG2, and upregulation of the differentiation marker GFAP (Supplemental Figure 3, A–C). Next, we leveraged available GSC-derived H3K27ac, SOX2, and OLIG2 ChIP-Seq data (34) and identified SOX2 and OLIG2 as potential drivers of KLHDC8A expression. OLIG2 and SOX2 displayed increased binding within 500 bp of the KLHDC8A superenhancer in GSCs (Figure 3C), suggesting that binding of these transcription factors at this superenhancer locus may drive the expression of KLHDC8A. Furthermore, KLHDC8A expression positively correlated with the expression of SOX2 and OLIG2 in glioblastoma patients from TCGA and CGGA databases (Figure 3D and Supplemental Figure 3D). Knockdown of SOX2 with 2 nonoverlapping shRNAs decreased mRNA expression of KLHDC8A (Figure 3E and Supplemental Figure 3, E and F). In single-cell RNA-Seq data from 28 glioblastoma patients, KLHDC8A was preferentially expressed in neuronal and neoplastic populations, and KLHDC8A expression overlapped with SOX2^+^ glioblastoma cells (Supplemental Figure 3, G and H). The expression of superenhancer-associated genes is mediated by the binding of transcriptional coactivators, prominently BRD4 (Bromodomain Containing 4). Inhibition of BRD4 leads to selective loss of the expression of superenhancer-driven genes (35). To validate that the expression of KLHDC8A was driven by a superenhancer, we treated GSCs with JQ1 — which preferentially inhibits BRD4 — in 2 GSC lines and observed downregulation of KLHDC8A mRNA expression in a concentration-dependent manner (Figure 3F). To interrogate the functional role of the predicted superenhancer locus in regulating KLHDC8A expression, we utilized a CRISPR-dCas9-KRAB system, a targetable repressive epigenetic factor that induces histone methylation and deacetylation (36), to selectively inhibit the predicted superenhancer locus. Inhibition of the predicted superenhancer region reduced KLHDC8A mRNA expression and GSC proliferation (Figure 3, G–I), supporting the essentiality of the noncoding superenhancer element and further orthogonal validation of the shRNA knockdown approach.

### KLHDC8A supported GSC growth through regulation of the extracellular matrix and receptor signaling.

**Figure 4:**
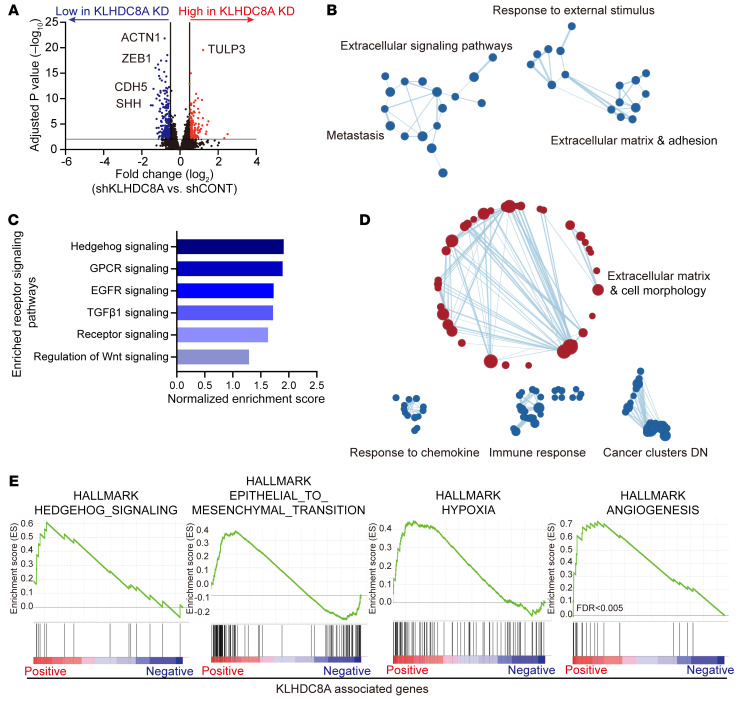
KLHDC8A promotes the expression of ECM and extracellular signaling genes. (**A**) Differentially expressed genes in GSC23 and GSC3028 transduced with shRNAs targeting KLHDC8A or a nontargeting control shRNA are displayed by volcano plot. Blue dots indicate genes downregulated in KLHDC8A knockdown cells at an adjusted *P* < 0.01 and log_2_ fold change less than −0.5. Red indicates genes upregulated following KLHDC8A knockdown at an adjusted *P* < 0.01 and log_2_ fold change greater than 0.5. (**B**) GSEA of GO pathways enriched or depleted following KLHDC8A knockdown in GSC23 and GSC3028 are displayed. Blue dots indicate enrichment in gene sets downregulated following KLHDC8A knockdown at an FDR < 0.15. (**C**) Top 6 downregulated receptor signaling signatures following KLHDC8A knockdown in GSC23 and GSC3028. Enriched gene signatures are plotted with normalized enrichment score. (**D**) Bubble plots showing the GSEA of gene sets positively or negatively correlated with KLHDC8A expression in TCGA glioblastoma HG-U133A data set. Blue dots indicate enrichment in gene sets negatively correlated with KLHDC8A expression. Red dots indicate enrichment in gene sets positively correlated with KLHDC8A expression. (**E**) GSEA of Hallmark gene sets correlated with KLHDC8A expression in TCGA glioblastoma HG-U133A data set are shown.

Little is known about the physiologic and pathologic functions of KLHDC8A in any tissue type or disease process. To elucidate the molecular mechanisms by which KLHDC8A promotes GSC growth, we performed RNA-Seq following KLHDC8A knockdown in 2 patient-derived GSCs, which revealed widespread gene expression changes when compared with a nontargeting control (Figure 4A) and altered expression of gene sets associated with extracellular matrix, cell adhesion, and extracellular stimulus response (Figure 4B and Supplemental Figure 4A). Among the top 6 downregulated receptor signaling signatures (Figure 4C), hedgehog signaling was the top downregulated signature following KLHDC8A depletion, suggesting a potential role of KLHDC8A in hedgehog signaling. In parallel, we leveraged clinical data sets to interrogate gene sets positively or negatively correlated with KLHDC8A expression. Similar to the results of RNA-Seq analysis, gene set enrichment analysis (GSEA) revealed that KLHDC8A expression positively correlated with gene sets associated with extracellular matrix, extracellular signaling, and cell morphogenesis. KLHDC8A-associated genes were negatively enriched for processes of chemokine response, immune response, and cancer clusters (Figure 4D). KLHDC8A-correlated genes strongly correlated with hedgehog signaling, angiogenesis, epithelial-to-mesenchymal transition, and hypoxia, which are modulated by signals from the extracellular environment and signals and are molecular processes associated with the progression of glioblastoma (Figure 4E). Collectively, these results implicate the potential roles of KLHDC8A in mediating extracellular signaling pathways, specifically in hedgehog signaling.

### KLHDC8A supports GSC growth via upregulation of hedgehog signaling.

**Figure 5:**
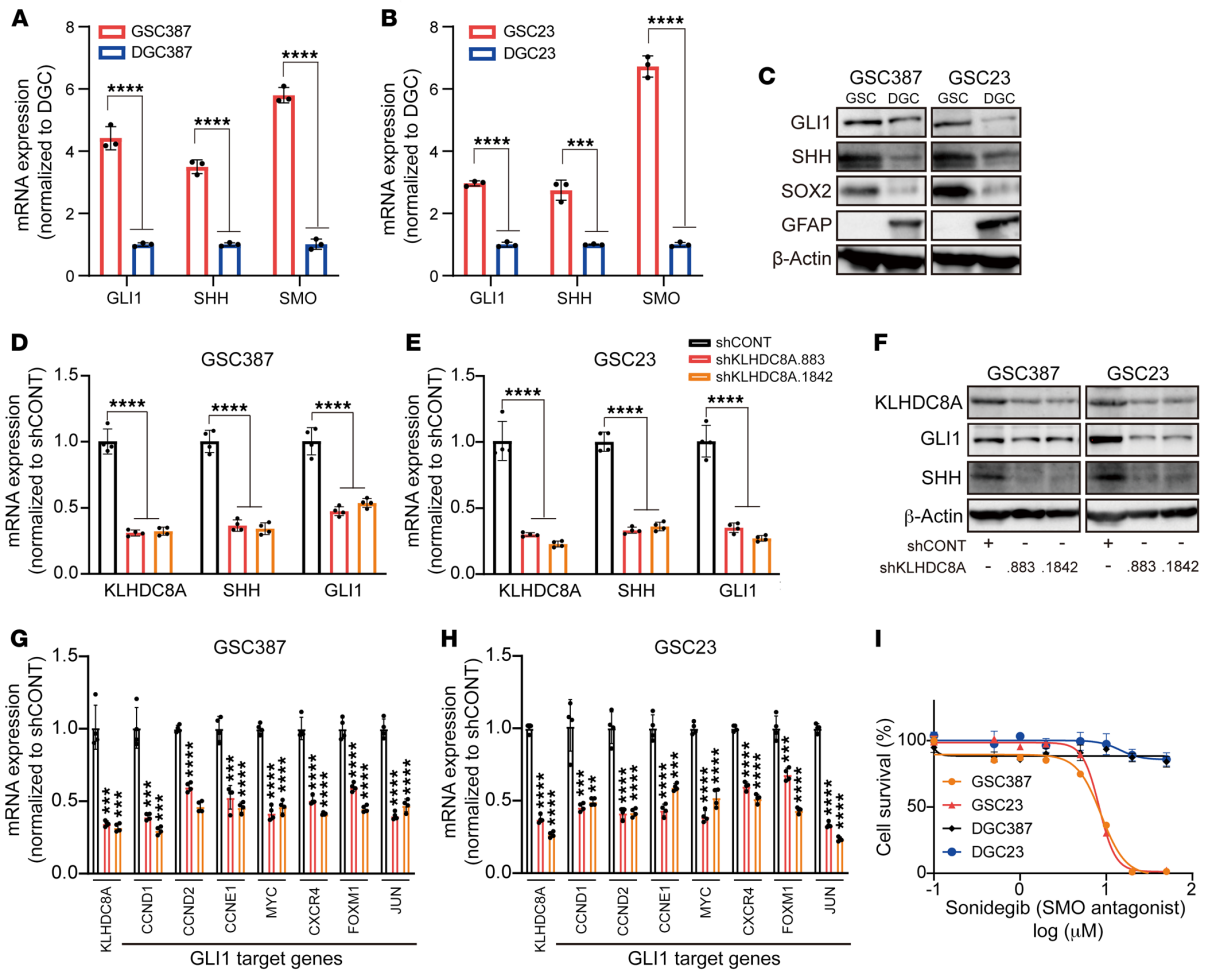
KLHDC8A promotes hedgehog signaling pathways in GSCs. (**A** and **B**) qPCR analysis of mRNA expression of hedgehog pathway genes SHH, SMO, and GLI1 in 2 GSCs and matched DGCs. *n* = 3. Quantitative data from 3 independent experiments are shown as mean ± SD. Statistical analysis was performed using Student’s *t* test with the Holm-Šidák multiple test correction. (**C**) Immunoblot showing the protein expression of Shh and Gli1 in 2 matched pairs of GSCs and DGCs is shown. SOX2 was used to determine the stemness of GSCs. GFAP was used to determine the differentiation of GSCs. β-Actin was used as the loading control. (**D** and **E**) qPCR analysis of KLHDC8A, SHH, and GLI1 mRNA expression in GSC387 and GSC23 following knockdown of KLHDC8A. *n* = 4. Quantitative data from 4 independent experiments are shown as mean ± SD. Statistical analysis was performed using 2-way ANOVA with the Šidák multiple test correction. (**F**) Immunoblot showing the protein expression of GLI1 and SHH upon KLHDC8A knockdown. (**G** and **H**) qPCR analysis of mRNA expression of GLI1 target genes in GSC387 (**G**) and GSC23 (**H**). *n* = 4. Quantitative data from 4 independent experiments are shown as mean ± SD. Statistical analysis was performed using Student’s *t* test with Dunnett’s multiple test correction. (**I**) Concentration-response curves of 2 matched pairs of GSCs and DGCs to SMO inhibitor Sonidegib over a 6-day time course. ***P < 0.001, ****P < 0.0001.

Following KLHDC8A knockdown and the subsequent RNA-Seq analysis, sonic hedgehog (SHH) was the top downregulated gene upon KLHDC8A perturbation (Supplemental Figure 4B). Single-sample GSEA (ssGSEA) of glioblastoma patients from the TCGA–glioblastoma multiforme (TCGA-GBM) data set revealed strong correlation between KLHDC8A expression, hedgehog signaling, and primary cilia, which is an organelle required for vertebrate hedgehog signal transduction in development and cancer (Supplemental Figure 4, C–E) (37). The hedgehog pathway drives maintenance and migration of cancer stem cells (18), including in GSCs (38), so we interrogated the role of KLHDC8A in hedgehog signal transduction in GSCs. To validate the functional importance of the hedgehog signaling pathway in our patient-derived GSCs, we analyzed the mRNA and protein levels of hedgehog pathway genes SHH and GLI1 in GSCs and DGCs. mRNA and protein levels of SHH and GLI1 were elevated in GSCs compared with DGCs, suggesting that hedgehog signaling may promote GSC growth (Figure 5, A–C). To assess the effect of KLHDC8A knockdown on hedgehog signaling, KLHDC8A was targeted with 1 of 2 nonoverlapping shRNAs, which revealed downregulation of SHH and the downstream effector, GLI1, at both mRNA and protein levels (Figure 5, D–F). Targeting KLHDC8A also reduced mRNA expression of several GLI1 target genes — including MYC, JUN, CXCR4, and FOXM1 — and cell cycle genes CCND1, CCND2, and CCNE1 (Figure 5, G and H). In complementary studies, GSCs and DGCs were treated with the SMO inhibitor, Sonidegib. GSCs were more vulnerable to SMO inhibition than DGCs (Figure 5I). These data suggest that KLHDC8A promoted GSC maintenance through upregulation of hedgehog signaling.

### KLHDC8A promoted primary cilia formation in GSCs.

**Figure 6:**
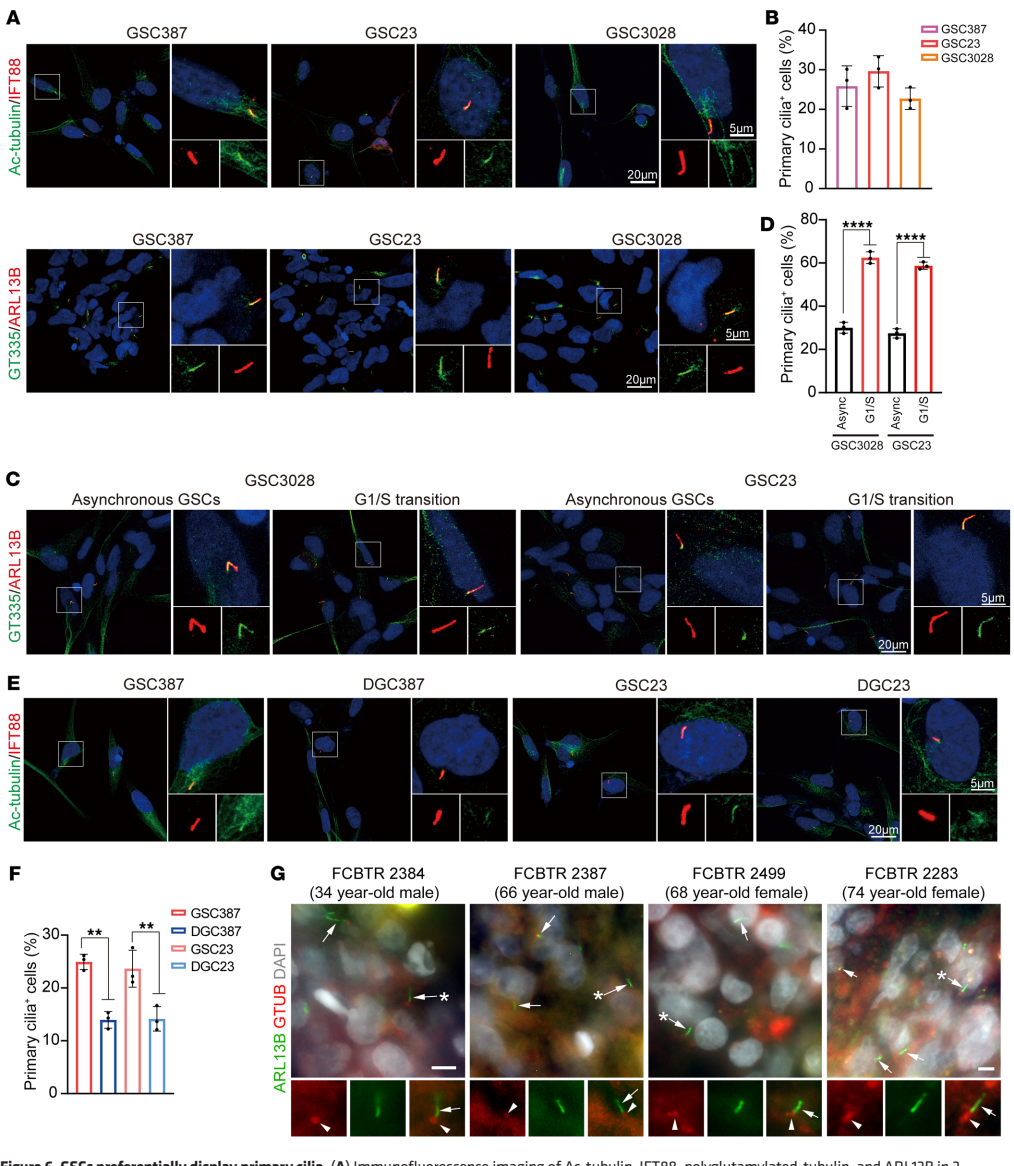
GSCs preferentially display primary cilia. (**A**) Immunofluorescence imaging of Ac-tubulin, IFT88, polyglutamylated-tubulin, and ARL13B in 3 patient-derived GSCs (GSC387, GSC23, GSC3028). Ac-tubulin or polyglutamylated-tubulin is shown in green, IFT88 or ARL13B in red, and DAPI in blue. (**B**) Quantification of primary cilia positive cells in GSC387, GSC23, and GSC3028. Data are presented as mean ± SD. At least 100 cells in each GSC line from 3 independent experiments were tested. (**C**) Immunofluorescence imaging of polyglutamylated-tubulin and ARL13B in patient-derived GSCs (GSC23 and GSC3028) following the double thymidine block. Polyglutamylated-tubulin is shown in green, ARL13B in red, and DAPI in blue. (**D**) Quantitative data are shown as mean ± SD. Statistical analysis was performed using 1-way ANOVA with Tukey’s correction for multiple comparisons. (**E**) Immunostaining of Ac-tubulin and IFT88 in 2 matched GSCs and DGCs (GSC387 and GSC23). Ac-tubulin is shown in green, IFT88 in red, and DAPI in blue. (**F**) Quantification of primary cilia positive cells in 2 matched GSCs and DGCs. At least 100 cells in each GSC line from 3 independent experiments were tested. Quantitative data are shown as mean ± SD. Statistical analysis was performed using 1-way ANOVA with Tukey’s multiple comparisons. (**G**) Epifluorescent images showing ARL13B+ cilia (green, arrows) with GTUB+ basal bodies (red, arrowheads) from patient glioblastoma biopsies from a 34 year old man, 66 year old man, 68 year old woman, and 74 year old woman. Arrows with asterisks indicate cilia enlarged below and separated by individual and merged channels. Scale bars: 5 or 20 μm. **P < 0.01, ****P < 0.0001.

**Figure 7:**
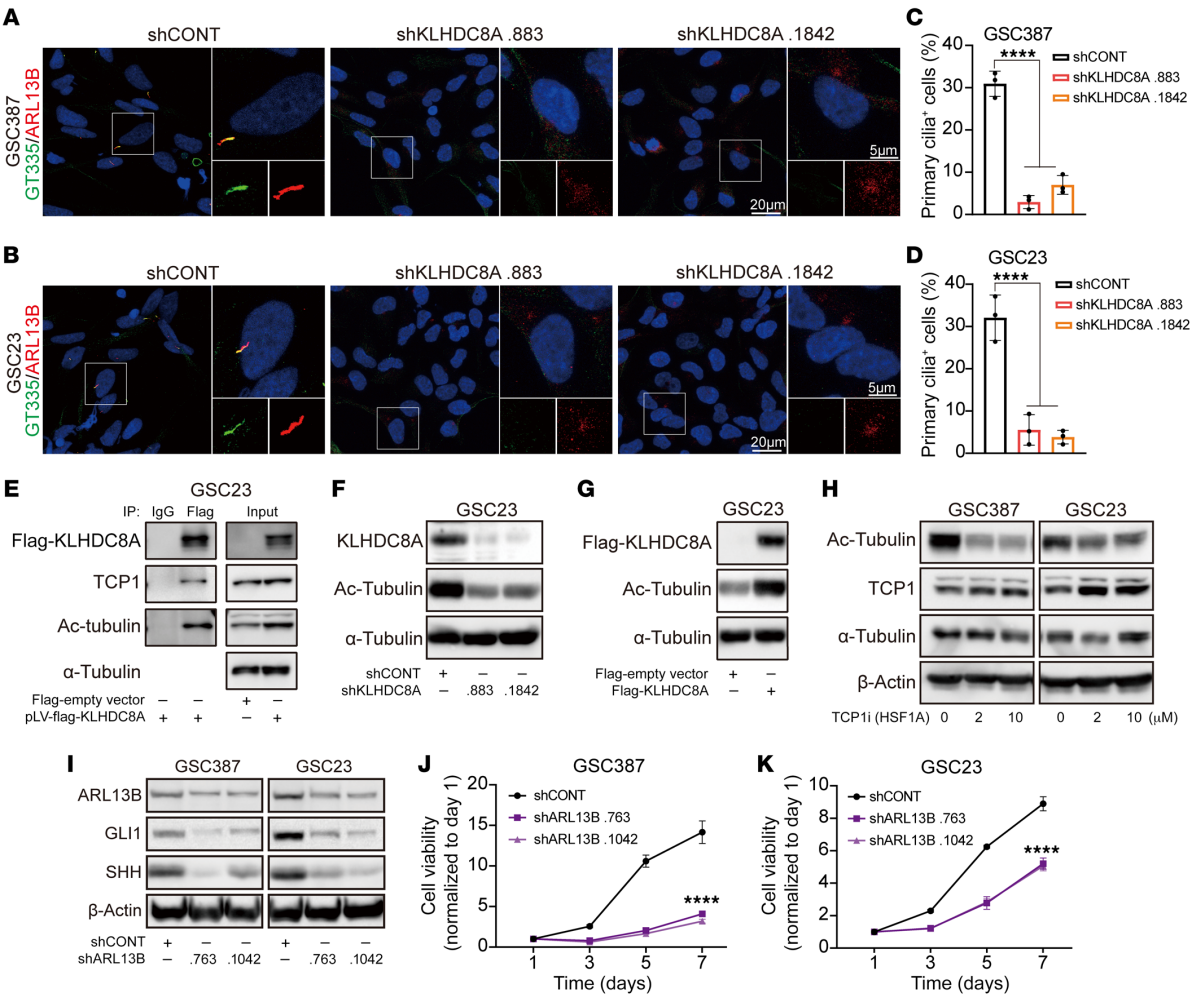
KLHDC8A is indispensable for primary cilia formation in GSCs. (**A** and **B**) Immunofluorescence imaging of primary cilia in GSC387 (**A**) and GSC23 (**B**) transduced with shCONT or 2 nonoverlapping shRNAs targeting KLHDC8A. Polyglutamylated-tubulin was labeled as green, ARL13B as red, and DAPI as blue. (**C** and **D**) Quantification of primary cilia positive GSC387 (**C**) and GSC23 (**D**). At least 100 cells in each GSC line from 3 independent experiments were tested. Quantitative data are shown as mean ± SD. Statistical analysis was performed using 1-way ANOVA with Tukey’s correction for multiple comparisons. (**E**) GSC23 cells were transduced with FLAG-KLHDC8A and then subjected to whole cell lysis. Coimmunoprecipitation for KLHDC8A was performed on the lysates with an anti-FLAG antibody or an IgG isotype control antibody. Immunoblotting was performed with anti-Flag, anti-TCP1, anti-Ac-tubulin, and anti-α-Tubulin antibodies. Inputs are indicated. (**F**) Immunoblot of the protein expression of Ac-tubulin following KLHDC8A knockdown is shown. (**G**) Immunoblot of the protein expression of Ac-tubulin following FLAG-KLHDC8A overexpression is shown. (**H**) Immunoblot showing the protein expression of Ac-tubulin, TCP1, and α-Tubulin following treatment of TCP1 inhibitor HSF1A. (**I**) Immunoblot showing the protein expression of ARL13B, Gli1, and Shh following ARL13B knockdown. (**J** and **K**) Cell viability in GSC387 (**J**) and GSC23 (**K**) over a 6-day time course following knockdown of ARL13B. *n* = 4. Quantitative data from 4 technical replicates are shown as mean ± SD. Statistical analysis was performed using 2-way ANOVA with Tukey’s multiple comparison. **** *P* < 0.0001. Scale bars: 5 or 20 μm.

Primary cilia are microtubule-based structures that function as cellular antennae, sensing and transducing mechanical, optical, or chemical signals in a cell type– and cell cycle phase–specific manner. Inhibition of primary cilia formation leads to loss of SHH-dependent ventral neural cell types (39). Primary cilia have been linked to glioma differentiation (40), so we investigated primary cilia in GSCs. Three patient-derived GSCs were stained with the primary cilia markers acetylated-α-tubulin (Ac-tubulin), IFT88, ARL13B, and polyglutamylated-tubulin, which label the axoneme of a cilium. Approximately 25% of GSCs displayed primary cilia detected by positive staining of Ac-tubulin, IFT88, ARL13B, and polyglutamylated-tubulin (Figure 6, A and B). Primary cilia formation is tightly regulated during cell cycle progression in dividing cells, with cilia present in G_1_ phase, usually in S phase, but generally resorbed in late G_2_ and completely disassembled prior to mitotic entry (41). To interrogate the relationship between ciliogenesis and cell cycle, we synchronized GSCs in the G_1_/S transition using a double thymidine block. We observed an increase in the percentage of primary cilia-positive cells in synchronized GSCs compared with asynchronous GSCs, showing that the presence of primary cilia in GSCs is controlled by cell cycle regulation (Figure 6, C and D). GSCs displayed a higher frequency of ciliated cells compared with matched DGCs (Figure 6, E and F), implicating a potential role of primary cilia in regulation of stemness. To interrogate whether primary cilia are associated with stemness, we transduced a SOX2 promoter reporter expressing EGFP into GSCs and separated GSCs into GFP^lo^ and GFP^hi^ expression via FACS (Supplemental Figure 5A). GFP^hi^ GSCs demonstrated a higher frequency of ciliated cells compared with GFP^lo^ GSCs (Supplemental Figure 5, B and C). The presence of primary cilia in glioblastoma tissues was confirmed by staining for ARL13B and γ-tubulin in biopsy specimens from patients with glioblastoma, which is in line with other studies indicating that glioblastoma tumors contain ciliated cells (42, 43) (Figure 6G). Furthermore, 4 out of 5 patient-derived tumoroids generated from glioblastoma biopsies also display primary cilia (Supplemental Figure 5, D and E). To determine if KLHDC8A participates in primary cilia formation, we depleted KLHDC8A in 2 patient-derived GSCs. KLHDC8A depletion reduced the percentage of ciliated cells (Figure 7, A–D and Supplemental Figure 5F). Therefore, we interrogated the Biological General Repository for Interaction Data sets (BioGRID), which contains 2 million biological interactions for more than 80 species. KLHDC8A interacted with the subunits of Chaperonin-Containing TCP1 (CCT) complex (Supplemental Figure 6A), which mediates actin and tubulin biogenesis and regulates posttranslational modification of α-tubulin (44). To confirm this predicted interaction, FLAG-tagged KLHDC8A was expressed in GSCs, as limited reagents for KLHDC8A exist. KLHDC8A coimmunoprecipitated with TCP1 and Ac-tubulin (Figure 7E). Decreased tubulin acetylation was observed upon KLHDC8A knockdown (Figure 7F). Reciprocally, overexpression of FLAG-KLHDC8A promoted tubulin acetylation in GSCs (Figure 7G). Treating GSCs with TCP1 inhibitor HSF1A decreased Ac-tubulin protein levels (Figure 7H), suggesting that KLHDC8A promoted cilia formation via upregulation ac-tubulin.

To confirm the role of KLHDC8A in promoting ciliogenesis, we interrogated the correlation between KLHDC8A expression and 2 ciliary proteins, IFT88 and ARL13B, in the CGGA data set. IFT88 is an intraflagellar transport protein and ARL13B is a regulatory GTPase; both are required for ciliogenesis and activation of canonical hedgehog signaling pathway in basal cell carcinoma (45) and medulloblastoma (46). KLHDC8A mRNA expression correlated with the IFT88 and ARL13B expression in glioblastoma tissues (Supplemental Figure 6B). ARL13B was upregulated in glioblastoma tissues compared with normal brain tissues (Supplemental Figure 6C). High expression of ARL13B was associated with poor patient prognosis in TCGA GBM Aglient-4502A and TCGA GBM-LGG data sets (Supplemental Figure 6, D and E), which was in line with previous studies (43, 47). Furthermore, shRNA-mediated knockdown of ARL13B phenocopied KLHDC8A knockdown, as shown by downregulation of SHH and GLI1, as well as reduced cell proliferation (Figure 7, I–K). Inhibition of primary cilia formation using a pharmacologic ciliogenesis inhibitor Ciliobrevin A downregulated SHH and GLI1 as well as the stemness markers OLIG2 and SOX2 (Supplemental Figure 6F), suggesting that primary cilia are associated with the stemness of GSCs. Collectively, these results suggested that KLHDC8A supports hedgehog signaling by promoting ciliogenesis in GSCs.

### Aurora B/C kinase inhibitor activity correlates with KLHDC8A and selects for primary cilia and hedgehog signaling.

**Figure 8:**
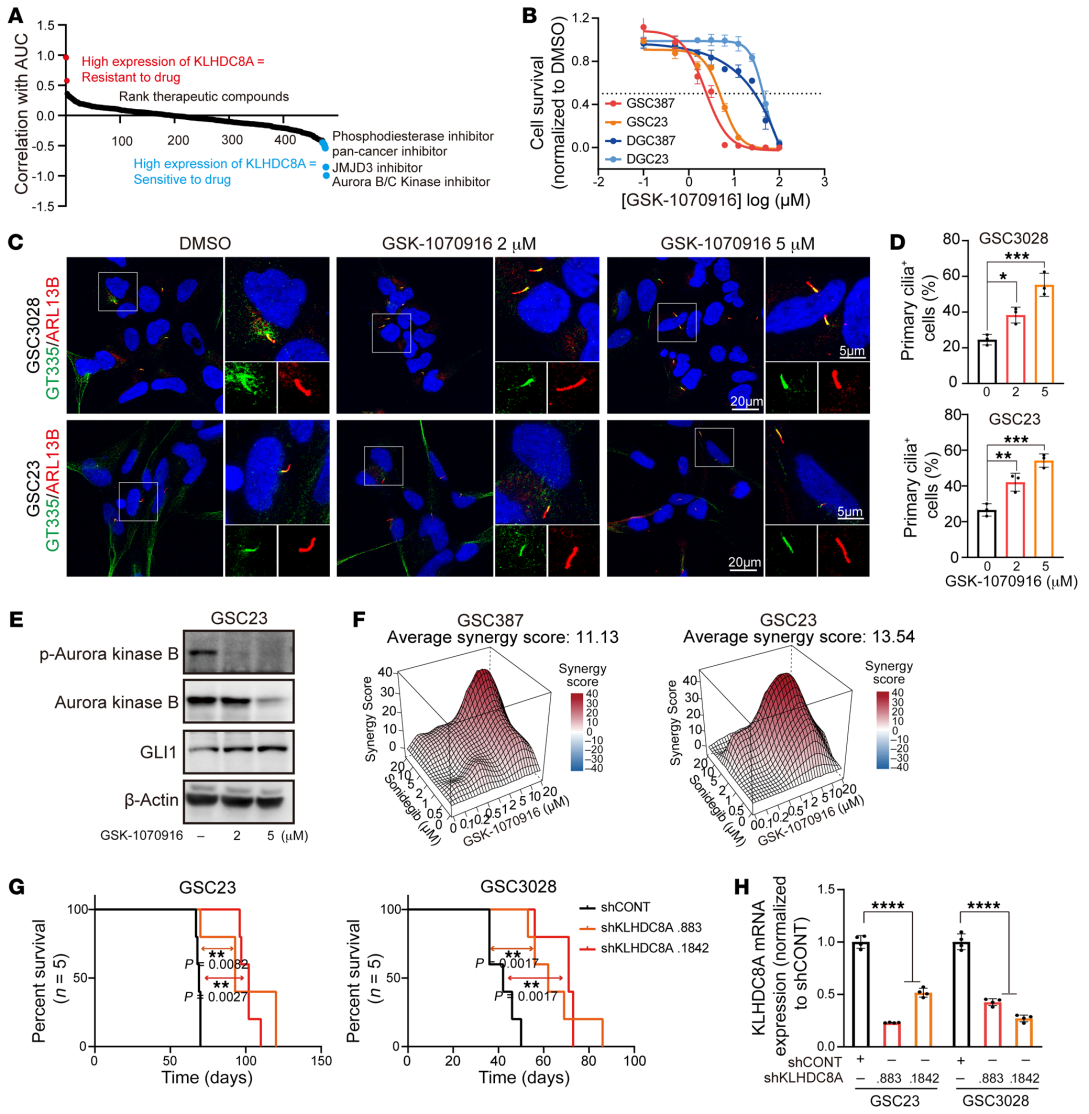
In vivo dependency and novel therapeutic strategies for targeting KLHDC8A in glioblastoma. (**A**) Plot showing ranked therapeutic compounds based on correlation of KLHDC8A mRNA expression with drug sensitivity (AUC) in brain cancer cell lines in CTRP data set. (**B**) Dose-response curves of 2 paired GSCs and DGCs to Aurora B/C kinase inhibitor, GSK1070916. (**C**) Immunofluorescence imaging of primary cilia in GSC3028 and GSC23 following treatment of GSK1070916. Polyglutamylated-tubulin was labeled as green, ARL13B as red, and DAPI as blue. (**D**) Quantification of cells possessing primary cilia GSC3028 and GSC23. At least 100 cells in each GSC line from 3 independent experiments were tested. Quantitative data are shown as mean ± SD. Statistical analysis was performed using 1-way ANOVA with Tukey’s correction for multiple comparisons. (**E**) Immunoblot showing the protein expression of phospho-Aurora kinase B, Aurora kinase B, and GLI1 following treatment of GSK1070916. (**F**) Synergy plots of Sonidegib and GSK1070916 in GSC387 and GSC23 analyzed by R package Synergyfinder. (**G**) Kaplan-Meier curve showing survival of NSG immunocompromised mice following implantation with GSC23 or GSC3028 following knockdown of KLHDC8A. *n* = 5 per group. Statistical analysis was performed using Mantel-Cox log-rank test. (**H**) The knockdown efficiency of KLHDC8A measured by qPCR in GSC3028 and GSC23. *n* = 4. Quantitative data from 4 independent experiments are shown as mean ± SD. Statistical analysis was performed using 1-way ANOVA with Tukey’s multiple comparisons. *P < 0.05, **P < 0.01, ***P < 0.001. Scale bars: 5 or 20 μm.

**Figure 9:**
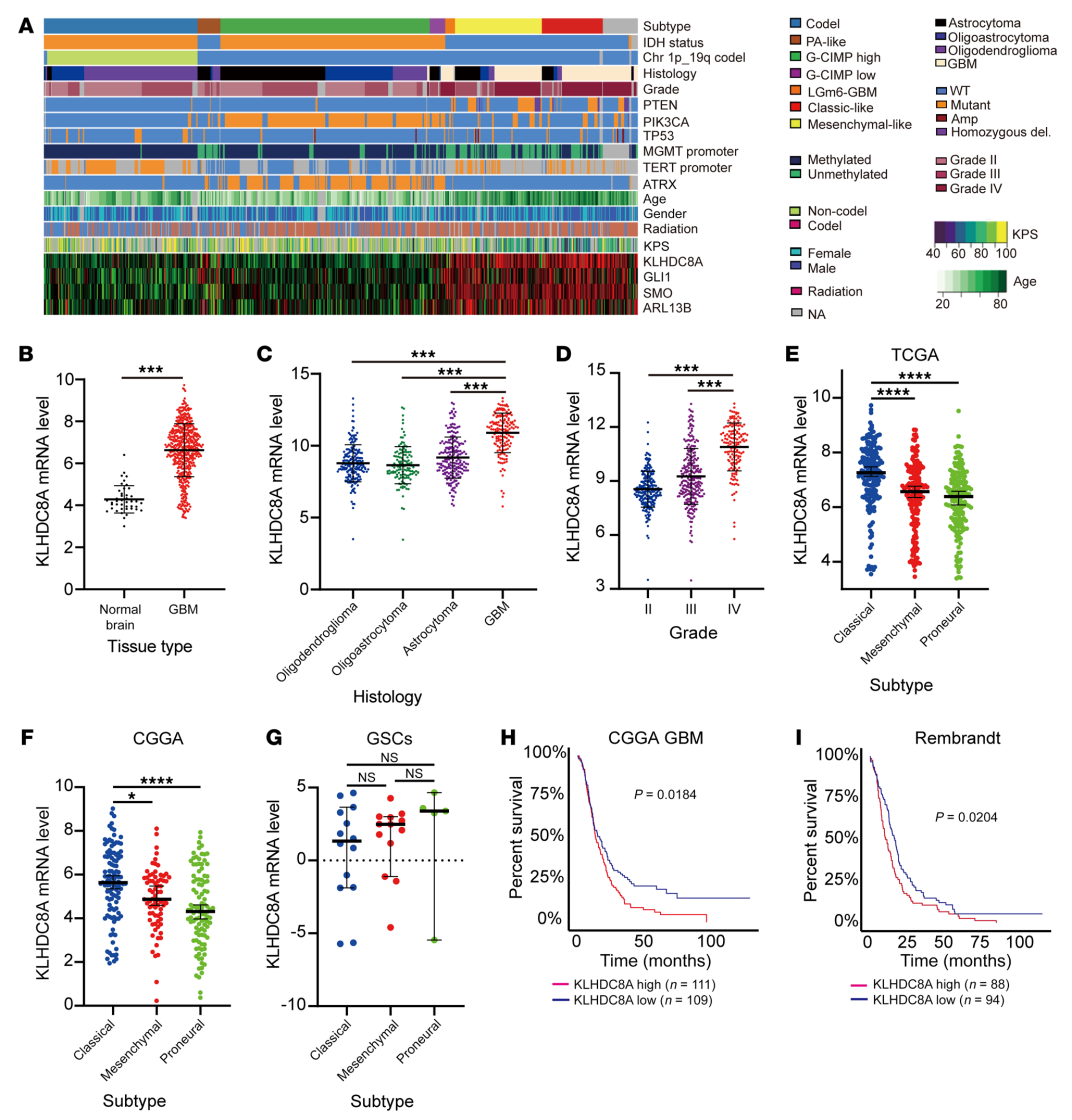
Clinical relevance of KLHDC8A. (**A**) Heatmap showing RNA-Seq, whole–exome-Seq, and clinical phenotype data along with KLHDC8A, GLI1, SMO, and ARL13B expression in each glioblastoma patient. (**B**) mRNA expression (TPM) of KLHDC8A in glioblastoma (*n* = 163) and normal brain (*n* = 21) in TCGA GBM data set. (**C** and **D**) KLHDC8A mRNA levels in different gliomas (**C**) or different grades (**D**) in TCGA of glioma. Data are presented as mean ± SD. Statistical analysis was performed using 1-way ANOVA with Tukey’s multiple comparisons. (**E**–**G**) KLHDC8A expression (TPM) in the molecular subtypes of glioblastoma tissues in (**E**) TCGA and (**F**) CGGA data sets and (**G**) patient-derived GSCs. Statistical analysis was performed using 1-way ANOVA with Tukey’s multiple comparisons. (**H** and **I**) Kaplan-Meier curves displaying survival of patients in CCGA GBM (**H**) and Rembrandt (**I**) stratified based on median mRNA expression of KLHDC8A. Data are presented as mean ± SD. Statistical analysis was performed using log-rank test. *P* = 0.0184 for CGGA GBM; *P* < 1e^–15^ for LGG-GBM; *P* = 0.0204 for Rembrandt. *P < 0.05, ***P < 0.001, ****P < 0.0001.

KLHDC8A lacks small-molecule binding pockets, rendering it potentially difficult to target. Therefore, we sought therapeutic dependencies correlated with KLHDC8A by leveraging the Cancer Therapeutics Response Portal (CTRP) and Cancer Cell Line Encyclopedia (CCLE) databases, which contain drug screening data of 481 small-molecule probes in 860 cancer cell lines and mRNA expression of 1,000 cancer cell lines, respectively. Elevated KLHDC8A expression was associated with sensitivity as measured by AUC with an Aurora B/C kinase inhibitor, a JMJD3 inhibitor, a pan-cancer inhibitor (BRD-4132) with unknown molecular targets, and an insulin-like growth factor 1 receptor (IGF1R) inhibitor (Figure 8A). Supporting the validity of this approach, we recently demonstrated that the IGF1R inhibitor, Linsitinib, targeted GSCs and displayed in vivo efficacy against glioblastoma xenografts (48). The Aurora B/C kinase inhibitor GSK1070916 has been tested in multiple human xenograft cancer types, including breast, colon, and lung, for its antitumor effects (49) and is in phase 1 clinical trials for solid tumors. As the Aurora B/C kinase inhibitor was the top hit, we speculated that GSCs, which display greater KLHDC8A expression levels, would be more vulnerable to inhibition of Aurora B/C kinases than DGCs. Indeed, Aurora B/C kinase treatment (GSK-1070916) inhibited GSC proliferation in a concentration-dependent manner, while DGCs were less sensitive at a concentration approximately tenfold greater than GSCs (Figure 8B). Aurora kinase A activation promotes primary cilia disassembly during G_1_ phase (50). However, the roles of Aurora kinase B and C in regulating primary cilia have not been explored. A previous study demonstrated that SHH-dependent medulloblastoma is sensitive to inhibition of pan-Aurora kinase inhibitor Danusertib (51), suggesting a potential interaction between primary cilia, hedgehog pathway, and Aurora kinase B and C. Therefore, we hypothesized that inhibition of Aurora B/C kinases may promote ciliogenesis and hedgehog signaling and that treatment with SMO inhibitor Sonidegib and Aurora B/C kinase inhibitor GSK-1070916 may exert combinatorial effects on GSCs. The frequencies of ciliated GSCs significantly increased after 24 hours of GSK-1070916 treatment (Figure 8, C and D). As revealed by immunoblotting, GLI1 was upregulated in cells treated with Aurora B/C kinase inhibitor, suggesting compensatory activation of hedgehog signaling (Figure 8E). Dual treatment with both inhibitors synergistically (Average synergy score of greater than 10) attenuated GSC proliferation (Figure 8F). In vivo tumor initiation is the gold standard assay for cancer stem cells. Thus, we interrogated whether disruption of KLHDC8A expression impaired in vivo tumor formation capacity. We intracranially implanted GSCs transduced with shCONT or 1 of 2 nonoverlapping shRNAs targeting KLHDC8A in immunocompromised mice. Mice bearing GSCs transduced with KLHDC8A shRNAs displayed prolonged survival compared with mice bearing shCONT GSCs (Figure 8, G and H). Expression of exogenous KLHDC8A in KLHDC8A-depleted GSCs restored in vivo tumor growth of GSCs (Supplemental Figure 7). To gain a clearer insight into the clinical relevance of KLHDC8A, we performed in silico analysis of TCGA data, revealing that KLHDC8A was preferentially expressed in glioblastoma tissues compared with normal brain tissues, and its expression, along with that of SMO, GLI1, and ARL13B, correlated with mesenchymal and classical subtypes, WT IDH tumors, high-grade glioma, and older patients (Figure 9, A–D). KLHDC8A expression was elevated in classical subtypes compared with mesenchymal and proneural subtypes (Figure 9, E and F). However, there was no significant difference in KLHDC8A expression between different molecular subtypes of GSCs (Figure 9G). KLHDC8A expression informed poor patient prognosis of patients in multiple brain tumor data sets (CGGA-GBM and Rembrandt) (Figure 9, H and I). Collectively, these data suggest that KLHDC8A is a regulator of hedgehog signaling and primary cilia formation and that targeting KLHDC8A through combinatorial SMO and Aurora B/C kinase inhibition is a promising therapeutic strategy for glioblastoma, serving as a potential therapeutic opportunity for targeting a previously undruggable target in glioblastoma.

## Discussion

Aberrant epigenetic dysregulation is an essential hallmark of many cancers (52). Superenhancers are enriched at genes that promote tumorigenesis in various cancer types, including medulloblastoma (53), colorectal cancer (54), leukemia (55), B cell lymphoma (56), and lung cancer (57). Pharmacological inhibitors or genetic ablation targeting key components of superenhancers and target genes impair the proliferation and in vivo tumor initiation capacity of cancer cells (15). Therefore, interrogation of superenhancers and their associated genes improve our understanding of GSC biology and allow for the identification of potential oncogenic drivers that promote tumorigenesis and progression.

Leveraging superenhancer profiling in glioblastoma tissues and GSCs, we identified an epigenetically upregulated gene, KLHDC8A, with expression driven by a superenhancer element located upstream of the gene with contributions from the stemness transcription factor SOX2. KLHDC8A belongs to a large family of kelch proteins, which generally contain 5–7 kelch tandem repeats and form a β-propeller tertiary structure known to mediate protein-protein interactions. Members of kelch proteins function through interaction with distinct binding partners and are involved in a wide range of cellular processes, including signal transduction (58), DNA repair (59), and protein degradation (60). The molecular functions of KLHDC8A have not been explored. In a previous study, KLHDC8A was highly expressed in glioblastoma cell lines that survived EGFR inhibitor treatment, and KLHDC8A compensated for the loss of a constitutively active variant of EGFR (EGFR VIII) (61). KLHDC8A is induced by lactate in glioblastoma cell lines (62). However, the mechanism by which KLHDC8A functions is unknown. Our study uncovers what we believe to be a novel function of KLHDC8A in promoting the hedgehog pathway through mediating ciliogenesis in GSCs.

Primary cilia are signaling hubs that host and mediate hedgehog signaling and other signaling pathways, including WNT, NOTCH, platelet-derived growth factor (PDGF), and various G-protein coupled receptors. However, the presence of primary cilia in glioblastoma tissues and patient-derived cell lines remains an area of investigation. Defects in primary cilia formation have been noted in glioblastoma biopsies and established glioblastoma cell lines (63, 64). We recently demonstrated that cilia induction promotes differentiation of a subset of cultured GSCs upon inhibition of Nek2 (40). However, substantial fractions of cells in glioblastoma biopsies and patient-derived human and mouse primary glioblastoma cells are ciliated with ultrastructural normal cilia (42). The distal tips of primary cilia on patient-derived glioblastoma cell lines secrete mitogenic vesicles and promote the proliferation of other ciliated glioblastoma cells (43). We found that approximately 20–25% of our patient-derived GSCs display primary cilia, which are in line with multiple studies that 10%–30% of glioblastoma tissues and cells are ciliated, and the presence of primary cilia in GSCs is tightly controlled during cell cycle progression. Furthermore, ARL13B knockdown and Ciliobrevin A treatment phenocopied the effect of KLHDC8A knockdown on hedgehog signaling, stemness, and proliferation, suggesting that primary cilia serve oncogenic roles in GSCs.

Mechanistically, we uncovered a novel function of the KLHDC8A in regulating tubulin biogenesis. KLHDC8A interacts with Ac-tubulin and the molecular chaperone CCT, which mediates tubulin biogenesis. Downregulation of KLHDC8A reduced Ac-tubulin expression, while KLHDC8A overexpression promoted tubulin acetylation in GSCs. Given that the β-propeller architecture mediates protein-protein interaction, we reasoned that KLHDC8A may function as an adaptor that facilitates CCT-α-tubulin interaction and subsequent tubulin folding and acetylation. In addition to regulating tubulin biogenesis, CCT is essential for Bardet-Biedl syndrome protein complex (BBSome) assembly, which exerts a pivotal role in primary cilia homeostasis by promoting cargo entry into cilia (65). Upregulated CCT is associated with enhanced proliferation and growth of breast cancer cells (66). Furthermore, elevated tubulin acetylation is linked to enhanced invasive migration and therapeutic resistance to chemotherapy agents (67, 68). Genetic ablation of α-tubulin acetyltransferase, αTAT1, suppresses colon cancer proliferation and invasion (69). Thus, the identification of novel KLHDC8A-CCT interaction may provide a further node of therapeutic benefit.

To identify a translational approach for targeting of KLHDC8A, we identified Aurora B/C kinases as the potential therapeutic targets in GSCs. GSCs display greater sensitivity to the inhibition of Aurora B/C kinase inhibitor compared to differentiated cells. GLI1 was upregulated upon treatment with Aurora B/C kinase inhibitor, and combined treatment of SMO inhibitor and Aurora B/C kinase inhibitor synergized to kill GSCs, indicating crosstalk between Aurora B/C kinase and the hedgehog pathway. Aurora kinase inhibitors are under development for the treatment of numerous cancers. Our results suggest that these inhibitors either select for cells with primary cilia and active hedgehog signaling or induce these states, supporting a likely molecular mechanism of therapeutic resistance. As Aurora kinase B is a mitotic kinase that regulates chromosome segregation and cell cycle progression during mitosis, dual treatment with a SMO inhibitor and Aurora B/C kinase inhibitor might target both ciliated and mitotic tumor cells. In conclusion, these findings demonstrate that KLHDC8A supports hedgehog signaling via upregulation of ciliogenesis. Dual treatment of hedgehog pathway and Aurora B/C kinase inhibitors may offer a novel therapeutic paradigm for treatment of glioblastoma.

## Methods

### Derivation of GSCs, nonmalignant brain cultures, and NSCs.

Patient-derived GSCs, GSC387, and GSC3028 were generated in our laboratory, as reported previously (7). GSC23 was obtained via a material transfer agreement from The University of Texas MD Anderson Cancer Center (Houston, Texas, USA; Supplemental Table 1). Patient-derived xenografts were generated and maintained as a recurrent source of tumor cells for study. Immediately upon xenograft removal, xenograft tumors were dissociated using a papain dissociation system (Worthington Biomedical Corp) according to the manufacturer’s instructions. Cells were cultured in Neurobasal medium supplemented with 2% B27, 1% L-glutamine, 1% sodium pyruvate, 1% penicillin/streptomycin, 10 ng/mL basic fibroblast growth factor (bFGF), and 10 ng/mL EGF for at least 6 hours to recover expression of surface antigens. No marker is uniformly informative for GSCs, so we used a combination of functional criteria to validate GSCs, including the expression of stem cell markers SOX2 and OLIG2, functional assays of self renewal, such as serial neurosphere passage, and tumor propagation using in vivo limiting dilution.

Nonmalignant brain cultures (NM176, NM177, and NM290) were derived in our lab from surgical resection specimens from patients undergoing surgery to treat epilepsy. Three nonmalignant neural stem/progenitor cell models — ENSA, hNP1, and NSC11 — were used in this study. ENSAs are human embryonic stem-derived neural progenitor cells (Millipore). NSCs are human-induced pluripotent-derived neural progenitor cells (ALSTEM). hNP1s are human iPSC-derived neural progenitors derived from the hESC WA09 line. All NSCs and GSCs were cultured in Neuroabasal media (Invitrogen) supplemented with B27 without vitamin (Invitrogen), sodium pyruvate, Glutamax, EGF, and bFGF (20 ng/mL each; R&D Systems). Differentiated glioblastoma cells derived from GSCs were generated and maintained in DMEM supplemented with 10% FBS for at least 7 days. Sphere formation assay, stemness markers SOX2 and OLIG2, and the differentiation marker GFAP were used to confirm the differentiation of DGCs. Short tandem repeat analyses were carried out annually to authenticate the identity of each tumor model used in this research. Mycoplasma testing was performed by quantitative PCR (qPCR) cellular supernatants at least once a year to ensure an absence of contamination. GSCs were passaged fewer than 20 times in vitro from xenografts.

### Staining of primary cilia in glioblastoma biopsies.

Immunofluorescent labeling of pathology-confirmed biopsies was performed as previously described (43, 70). Briefly, specimens were fixed within 1 hour of resection in either 4% paraformaldehyde in 0.1 M PBS or ice-cold methanol. Samples were rinsed in 1 × PBS, cryoprotected in 30% sucrose in 1 × PBS followed by a 1:1 solution of 30% sucrose in PBS and OCT (Thermo Fisher Scientific), frozen in OCT over liquid nitrogen and cryosectioned at 16 μm. Samples were incubated in blocking solution containing 5% normal donkey serum (NDS) (Jackson Immunoresearch) and 0.2% Triton-X 100 in 1 × PBS for 1 hour and incubated in primary antibodies with 2.5% NDS and 0.1% Triton-X 100 in 1 × PBS overnight at 4°C. Primary antibodies included rabbit-anti-ADP ribosylation factor 13B (ARL13B) (1:3000; Proteintech; 17711-1-AP), and mouse-anti-gamma-tubulin (1:3000; Sigma-Aldrich; T6557). Appropriate fluorescein (FITC) AffiniPure Goat Anti-Rabbit IgG (H+L) (1:1000; Jackson Immunoresearch; 111-095-045) and Cy™3 AffiniPure Goat Anti-Mouse IgG (H+L) secondary antibodies (1:1000; Jackson Immunoresearch; 115-165-003) in 2.5% NDS with 1 × PBS were applied for 2 hours at RT. Slides were coverslipped in Prolong Gold antifade media containing DAPI. Slides were examined under epifluorescence with an inverted Zeiss AxioObserver D1 microscope using a Zeiss 40 ×/0.95 plan Apochromat air objective or a Zeiss 63 ×/1.4 plan Apochromat oil objective. Images were captured and analyzed using Zeiss ZEN software.

### Generating patient-derived tumoroids.

The human glioma tumor tissues cultured in this study were collected by collaborating surgeon Michael Sabel at the Universitätsklinikum Düsseldorf (Düsseldorf, Germany) and immediately collected for processing at the Laboratory for Centrosome and Cytoskeletal Biology. Patient-derived tumoroids were generated based on Jacobs et al. (71) with slight modifications. In brief, collected tumors tissues were washed with PBS to remove debris and blood cells and manually chopped into 200–500 μm pieces with a sterile razor blade in a glass dish containing GBO medium. The chopped tissues were transferred into a low-attachment cell culture dish containing GBO medium and incubated at 37°C with 5% CO_2_ for 24 hours before being transferred to an orbital shaker (10 *g*) for long-term culturing.

### Immunofluorescent staining.

Cells were grown on coverslips coated with Matrigel hESC-Qualified Matrix (Corning). After treatments, cells were fixed with 4% paraformaldehyde in PBS at room temperature for 10 minutes, followed by permeabilization with PBS containing 0.5% Triton X-100 at room temperature for 15 minutes, and blocked with PBS containing 5% goat serum and 0.1% Triton X-100 at room temperature for at least 1 hour. Cells were incubated with desired primary antibodies overnight at 4°C. All antibodies used in this study are listed in Supplemental Table 2. Cells were washed with PBS and incubated with Fluorescent-dye conjugated secondary antibodies (1:500; Sigma-Aldrich) at room temperature for 1 hour. Cells were washed with PBS and incubated with PBS supplemented with DAPI at room temperature for 5 minutes. After the final wash with PBS, slides were mounted with ProLong Diamond Antifade Mountant (Thermo Fisher Scientific) and imaged with Leica SP8 CLARITY confocal microscope. Images were captured using 20 × air or 100 × oil objectives. The interval between the Z stacks was kept 0.5 μm apart. Images were processed using Leica Application Suite X software and Adobe Illustrator CS6.

### Western blotting.

Cells were lysed in RIPA buffer supplemented with protease inhibitors and phosphatase inhibitors on ice for 30 minutes. Lysates were centrifuged at 4°C for 15 minutes at 14,000*g* and the supernatants were collected. The concentration of lysates was determined by utilizing the bicinchoninic acid (BCA) assay according to the manufacturer’s protocol. Equal amounts of protein samples were mixed with 6 × SDS Laemmli loading buffer and were boiled for 10 minutes. The protein samples were used directly for SDS-PAGE electrophoresis or stored at –80°C. The PVDF membranes with proteins were blocked with 5% nonfat milk in TBST at room temperature for 1 hour, followed by incubation with primary antibodies overnight at 4°C. All the antibodies used in this study are listed in Supplemental Table 2. The membranes were incubated with desired secondary antibodies and were developed by SuperSignal West Pico Plus Chemiluminescent Substrate (Thermo Fisher Scientific) and were imaged using BioRad image lab software. See complete unedited blots in the supplemental material.

### Plasmids and lentiviral transduction.

Lentiviral vectors expressing 2 nonoverlapping shRNAs targeting KLHDC8A, SOX2, ARL13B, or a control shRNA that does not target any known mammalian genes were obtained from Sigma-Aldrich. All shRNAs used in this study were listed in Supplemental Table 3. For lentivirus production, 293FT cells were cotransfected with lentiviral plasmids bearing shRNA sequences, viral packaging vectors pCMV-dR8.2, and VSVG using LipoD293 in vitro DNA transfection reagent (SignaGen) in DMEM supplemented with 10% FBS according to the manufacturer’s protocol. Culture medium was replaced with complete DMEM medium 72 hours after transfection. Medium containing virus particles was collected, and the supernatant was filtered with a 0.45-μm filter. Lenti-X Concentrator (TaKaRa Cat# 631232) was used to concentrate the virus according to the manufacturer’s protocol. The virus particles were resuspended in complete Neurobasal medium and were used immediately or stored at −80°C.

### Quantitative reverse-transcription PCR.

Total cellular RNA was isolated using Trizol reagent (Invitrogen) and Direct-zol RNA Miniprep Kits (Zymo Research) according to the manufacturer’s instructions. The RNA was eluted and dissolved in RNase-free water and was subsequently reversed transcribed by using a cDNA Reverse Transcription kit (Life Technologies). All cDNAs were reverse transcribed from 1 μg total RNA. Quantitative reverse-transcription PCR was performed using Applied Biosystems 7900HT cycler with Radiant Green Hi-ROX qPCR kit (Alkali Scientific).

### Apoptosis assay.

Apoptosis was assessed using FITC Annexin V Apoptosis Detection Kit I (BD Biosciences) according to the manufacturer’s protocol. Briefly, cells were harvested and washed twice with ice-cold PBS. Appropriated numbers of cells were incubated in 100 μl Annexin-binding buffer containing FITC annexin V and Propidium iodide (PI) at room temperature in the dark for 15 minutes. A total of 400 μl Annexin-binding buffer was added to each sample. The samples underwent flow cytometry analysis using a BD LSR Fortessa Flow Cytometer.

### Isolation of GFP^hi^ and GFP^lo^ GSCs.

FACS was performed to obtain GFP^hi^ and GFP^lo^ GSC3028 and GSC23. Briefly, GSC3028 and GSC23 were transduced with a SOX2 promoter reporter expressing EGFP and were dissociated into single cells. Cells were washed twice with PBS and resuspended in cell staining buffer (BioLegend) followed by FACS using a Beckman Coulter MoFlo Astrios.

### Proliferation and neurosphere formation assays.

Cells were plated at a density of 2,000 cells per well in a 96-well plate with at least 4 replicates. Cell viabilities were assessed at desired time points using CellTiter-Glo (Promega) assay according to the manufacturer’s protocol. Cell viabilities were normalized to the data of day 0 and were presented as mean ± SD. Neurosphere formation of cells was assessed by in vitro limiting dilution assay. Briefly, different numbers of cells (50, 20, 10, and 1) were plated into 96-well plates with 24 replicates. The presence and number of neurospheres in each well were counted 7 days later after plating. The frequencies of stem cells were analyzed using software available at http://bioinf.wehi.edu.au/software/elda, as previously described.

### H3K27aC Chip-Seq analysis.

H3K27ac Chip-Seq data for glioblastoma tissues and GSCs were accessed and downloaded through GSE101148, GSE72468, and GSE119834. Single-end fastq files of H3K27ac and Input Chip-Seq reads were first trimmed to get rid of adaptor sequence using “Trim Galore!” and “Bowtie2” was used to align ChIP-Seq reads to hg19 reference genome. The output SAM files were converted to BAM files and sorted by genomic coordinates using “SAMtools” and “Sambamba”. H3K27ac peaks were called using “MACS2” using ChIP input files as controls with a q value cutoff of 0.001. To visualize the enrichment patterns at particular locations in the genome and evaluate regions of differential enrichment, Bigwig files were generated from BAM files using “DeepTools” 3.5.0 BamCoverage. Superenhancers in glioblastoma tissues and GSCs were identified by using the Rank-Ordering of Super-Enhancers (ROSE) algorithm on hg19 human genome. ROSE was performed with a stitching distance of 12,500 bp and a transcriptional start site exclusion distance of 2,500 bp. Superenhancers were ranked by counting the H3K27ac signal in the ChIP file compared with the matched input file. De novo motifs were called from GSC enhancer regions within glioblastoma tissue superenhancers using Hypergeometric Optimization of Motif EnRichment (HOMER) “findmotifsgenome.pl” script. Top scoring enriched motifs and transcription factors were presented.

### In vitro drug studies and synergy calculations.

For In vitro cell viability assay, 5,000 cells were plated in each well of 96-well plates with at least 5 replicates. Cells were treated with DMSO or desired drugs at multiple concentrations for 72 hours. Cell viabilities were assessed using CellTiter-Glo (Promega) assay according to the manufacturer’s protocol. For synergy calculations, synergy indices of aurora kinase B inhibitor and sonidegib were analyzed by R package Synergyfinder.

### Coimmunoprecipitation assay.

GSCs expressing FLAG-KLHDC8A were collected and washed with ice-cold PBS. Cell pellets were resuspended in Pierce IP Lysis Buffer (Thermo Fisher Scientific) supplemented with protease and phosphatase inhibitors and were placed on ice for 30 minutes. Cell lysates were centrifuged at 14,000*g* for 15 minutes at 4°C and the supernatant was harvested. The concentration of lysates was determined by utilizing the BCA assay according to the manufacturer’s protocol. A total of 1mg of cell lysate was mixed with anti-FLAG M2 magnetic beads (Sigma-Aldrich) and rotated at 4°C overnight. The beads were washed with Pierce IP lysis buffer 3 times and were boiled in 2X SDS Laemmli loading buffer at 95°C for 5 minutes. The supernatant was collected and used for Western blot analysis.

### RNA-Seq analysis.

RNA-Seq data for GSC versus DGC comparisons were accessed and downloaded from Gene expression Omnibus (GEO) with accession number GSE547391. 3 pairs of matched GSCs and DGCs (MGG4, MGG6, and MGG8) were used for this study. Counts-per-million and differential expression were calculated using Limma in R. Differentially expressed genes were filtered with the cutoff of log_2_ mRNA expression fold change of greater than 2 (or less than −2) and with an adjusted *P* value of less than 1 e^–3^.

For RNA-Seq analysis upon KLHDC8A knockdown, total cellular RNAs from GSCs transduced with shCONT or shKLHDC8A were isolated by Trizol reagent (Invitrogen) and Direct-zol RNA Miniprep Kits (Zymo Research) according to the manufacturer’s instructions. The purified RNA was eluted and dissolved in RNase-free water and was subsequently subjected to RNA-Seq. The Fastq files of RNA-Seq data were processed to read quality control by performing “FastQC”, and the adaptor sequences were removed by running “TrimGalore”. The trimmed reads were mapped to hg19 human genome and were counted by using Salmon in the quasi-mapping mode. Salmon output files were converted using Tximport, and differential expression analysis was performed using R package DESeq2. Volcano plots displaying differentially expressed genes was generated using GraphPad Prism. A prerank gene list was generated by selecting differentially expressed genes (FDR-corrected p value of less than 0.05). GSEA was performed by inputting the preranked gene list into GSEA desktop application. Bubble plot was generated using Cytoscape. All original RNA-Seq data on KLHDC8A knockdown from GSCs were deposited in the NCBI’s Gene Expression Omnibus (GEO) database (GEO GSE207760).

### In vivo tumorigenesis.

Intracranial xenograft assays were generated by injecting 10,000 GSCs transduced with shCONT or shKLHDC8A into the right cerebral cortex of NSG (NOD.Cg-*Prkd*c^scid^ *Il2rg*^tm1Wjl^/SzJ; The Jackson Laboratory) mice at a depth of 3.5 mm. A total of 5 mice per arm were used to generate the survival curves of mice bearing shCONT GSCs and shKLHDC8A GSCs. At least 4–6 week-old healthy, WT male or female mice of NSG background were selected in this study. All mice were monitored every day until neurological signs, including hunched posture, gait changes, lethargy, and weight loss, were observed, at which point they were sacrificed.

### Statistics.

All statistical analyses are described in the figure legends. For qPCR analysis, 2-tailed Student’s *t* test was performed to assess the statistical significance between 2 groups. 2-way ANOVA with Dunnett multiple hypothesis test correction was used for statistical analysis when appropriate. For mice survival analysis, Kaplan-Meier survival curves were generated by using GraphPad Prism 9 software, and the statistical significance between different groups was calculated by using log-rank tests. For cell proliferation assay, 2-way ANOVA with Dunnett multiple hypothesis test correction was applied for statistical analysis. For extreme limiting dilution assay, χ^2^ test was used for pairwise differences in assessing the frequencies of stem population.

### Study approval.

Surgically resected tumor biopsies from patients were collected and deidentified by the Florida Center for Brain Tumor Research (FCBTR) (University of Florida, Gainesville, Florida) and processed in accordance with University of Florida Institutional IRB protocol 201902489. All anonymized tissue samples were obtained with informed consent and were collected using protocols approved by the ethics committee of the University Hospital of Düsseldorf (2018-273-andere Forschung erstvotierend “Molekulare Analyse und Etablierung eines Zellkultur-Krankheitsmodells des Glioblastoms”). All murine experiments were performed under an animal protocol (s17096) approved by IACUC at the UCSD.

## Author contributions

DL conceptualized and designed the study, acquired and analyzed the results, and wrote the manuscript. RCG analyzed RNA-Seq data and revised the manuscript. BCP analyzed and interpreted the bioinformatics data. ZQ and XW analyzed the bioinformatic data and provided experimental assistance of animal experiments. QW provided experimental assistance of intracranial injections. MRS, JG, and VD revised the manuscript and provided technical support. DRR and JNR designed the study, wrote and revised the manuscript, and provided study supervision and administrative and material support.

## Supplementary Material

Supplemental data

## Version 1 11/17/2022

In-Press Preview

## Version 2 01/17/2023

Electronic publication
