# Supplementary material for: Superenhancer activation of KLHDC8A drives glioma ciliation and hedgehog signaling
Source: J Clin Invest. 2023 Jan 17;133(2):e163592. doi: 10.1172/JCI163592 (PMC9843063; doi:10.1172/JCI163592)

SUPPLEMENTAL FIGURES

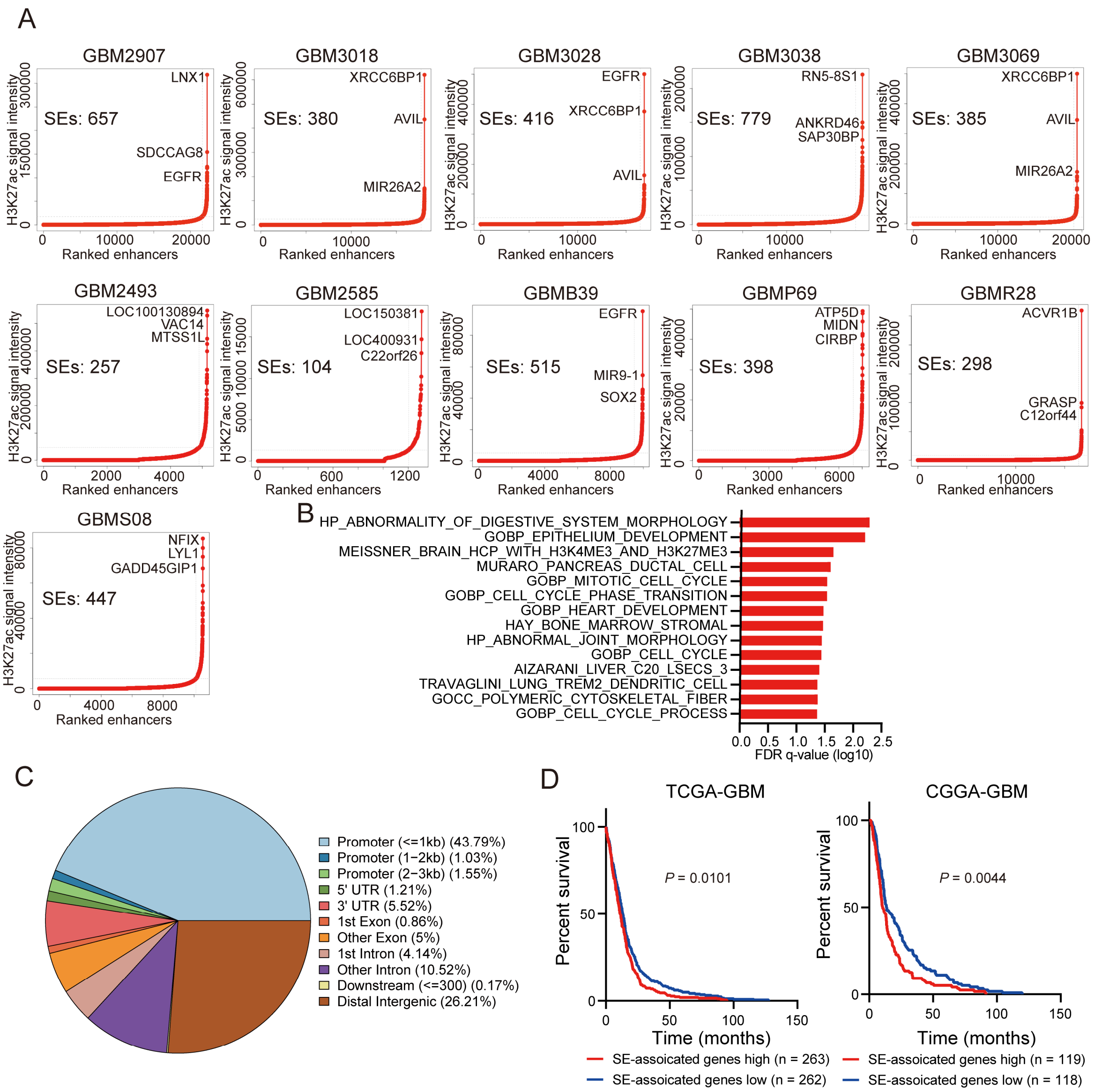

Supplemental Figure 1. In silico superenhancer screen identifies GSC superenhancer-associated targets, related to Figure 1.
(**A**) Hockey stick plot showing all superenhancer-associated genes from 11 glioblastoma tissues (GBM2907, 3018, 3028, 3038, 3069, 2493, 2585, B39, P69, R28, and S08). Superenhancers were identified by ROSE algorithm and were based on H3K27ac ChIP-seq data and the corresponding input ChIP-seq data. (**B**) Bar plot showing top 14 gene sets enriched among 252 selected GSC superenhancer-associated genes, as described in **B**. (**C**) Genome-wide annotations of selected GSC superenhancers, as described in **B**. (**D**) Kaplan-Meier curves displaying survival of patients in TCGA HG-U133A glioblastoma dataset and CGGA glioblastoma dataset stratified based on the signature score of selected GSC superenhancer-associated genes. Statistical analysis was performed using log-rank test. *P* = 0.0101 for TCGA GBM dataset and *P* = 0.044 for CGGA GBM dataset.

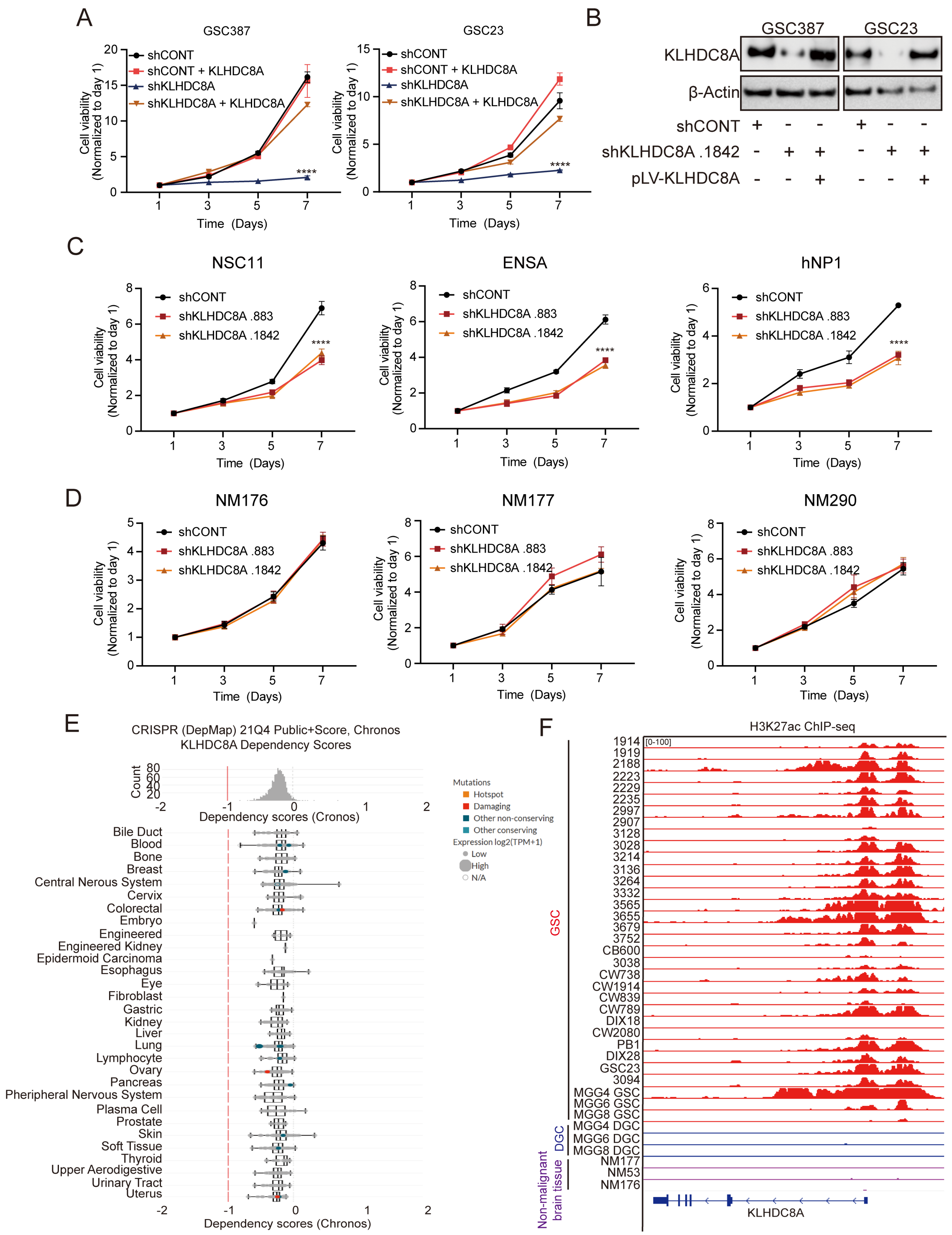

Supplemental Figure 2. KLHDC8A is necessary for stem population and is a strongly selective gene across cancer types, related to Figure 2. (**A**) Cell viability was measured by CellTiter-Glo assay in shCONT, shKLHDC8A, and KLHDC8A-rescued GSC387 and GSC23 over a 6-day time course following KLHDC8A knockdown. n=4. Quantitative data from 4 technical replicates are shown as mean ± SD (error bars). Statistical analysis was performed using two-way ANOVA with Dunnett's multiple comparisons. ****P<0.0001. (**B**) Protein levels of KLHDC8A following KLHDC8A knockdown and rescued were measured by immunoblot. β-Actin was used as the loading control. (**C**) Cell viability measured by CellTiter-Glo assay in 3 neural stem/progenitor cells (ENSA, hNP1, and NSC11) over a 6-day time course following knockdown with a non-targeting control shRNA or two non-overlapping shRNA targeting KLHDC8A (shKLHDC8A.883 or shKLHDC8A.1842). *n*=4. Quantitative data from 4 technical replicates are shown as mean ± SD (error bars). Statistical analysis was performed using two-way ANOVA with Dunnett's multiple hypothesis test correction. **** *P* < 0.0001. (**D**) Cell viability measured by CellTiter-Glo assay in three nonmalignant brain cells derived from epilepsy tissue resection specimens (NM176, NM177, and NM290) over a 6-day time course following knockdown with a non-targeting control shRNA or two non-overlapping shRNA targeting KLHDC8A. *n*=4.

Quantitative data from 4 technical replicates are shown as mean ± SD (error bars). (**E**) KLHDC8A dependency score in a whole-genome CRISPR-Cas9 screen across 558 cancer cell lines. A lower score means that a gene is more likely to be dependent in a given cell line. A score of 0 indicates a gene that is not essential, whereas a score of -1 corresponds to the median of all pan-essential genes. Data were derived from the Cancer Dependency Map (www.depmap.org). Dependency score is calculated using the Chronos algorithm. (**F**) H3K27ac signal at the KLHDC8A locus across an overlay of 33 GSCs, 3 DGCs, and 3 NM cells.

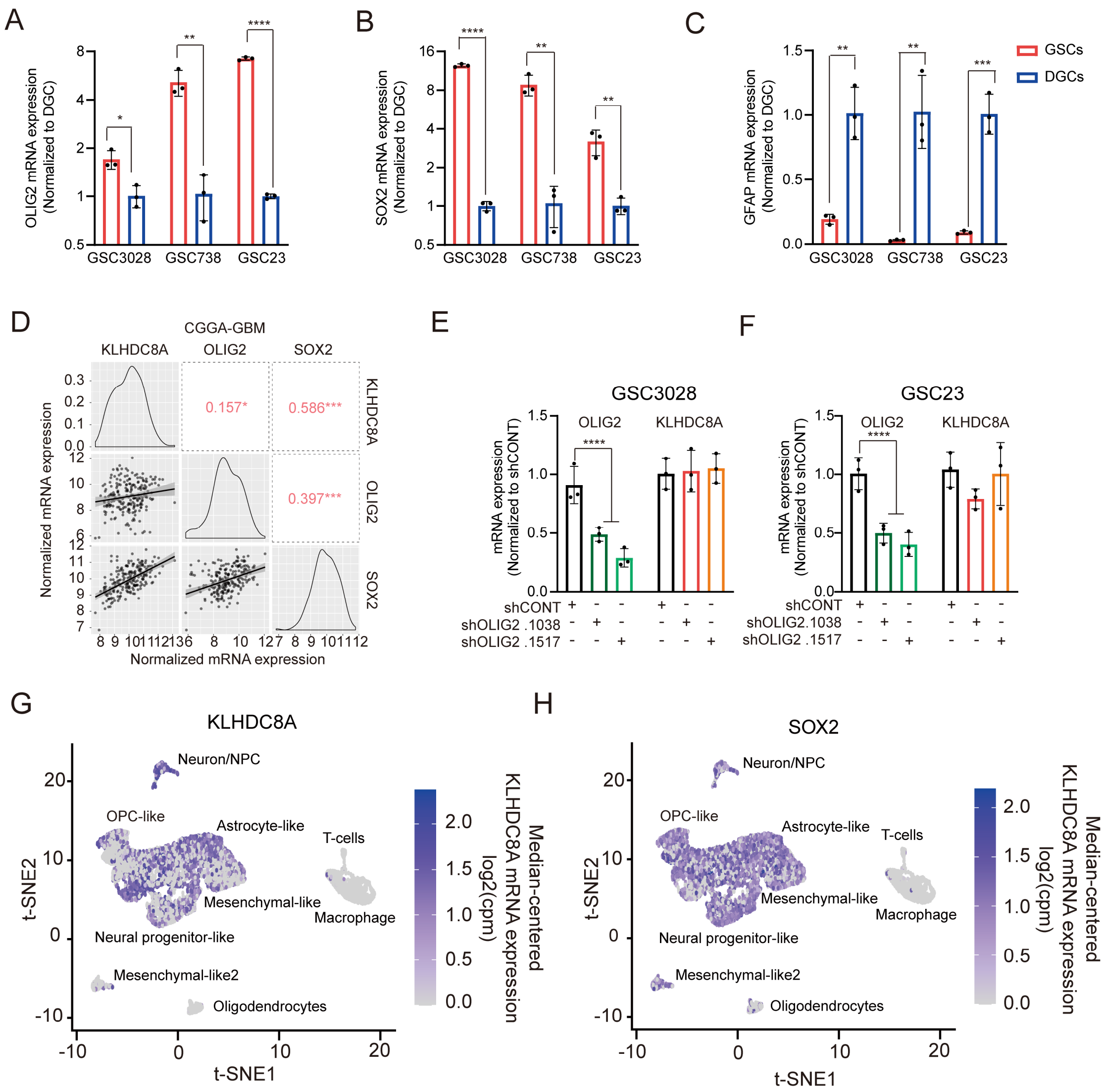

Supplemental Figure 3. KLHDC8A expression is preferentially expressed in GSCs and is driven by stem state transcription factor SOX, related to Figure 3.

(**A**) mRNA expression of OLIG2 measured by qPCR in three matched GSCs (3028, 738, and GSC23) and DGCs. *n*=3. Quantitative data from 3 independent experiments are shown as mean ± SD (error bars). Statistical analysis was performed using Student's t-test. ** $P < 0.01$, **** $P < 0.0001$. (**B**) mRNA expression of SOX2 measured by qPCR in three matched GSCs and DGCs. *n*=3. Quantitative data from 3 independent experiments are shown as mean ± SD (error bars). Statistical analysis was performed using Student's t-test. ** $P < 0.01$, **** $P < 0.0001$. (**C**) mRNA expression of GFAP measured by qPCR in three matched GSCs and DGCs. *n*=3. Quantitative data from 3 independent experiments are shown as mean ± SD (error bars). Statistical analysis was performed using Student's t-test. ** $P < 0.01$, *** $P < 0.001$. (**D**) Correlation of mRNA expression between KLHDC8A, OLIG2, and SOX2 in CGGA dataset. Numbers indicated the R-value of Spearman correlation. ** P < 0.01, *** $P < 0.001$. (**E** and **F**) qPCR analysis of mRNA expression of OLIG2 and KLHDC8A upon knockdown of OLIG2. Statistical analysis was performed using two-way ANOVA with the Sidak multiple test correction. **** $P < 0.0001$. (**G**) t-SNE plot demonstrating expression of KLHDC8A across cell types. Data are presented as median-centered mRNA expression in counts per million (CPM). (**H**) Similar analysis was performed for SOX2 mRNA expression.

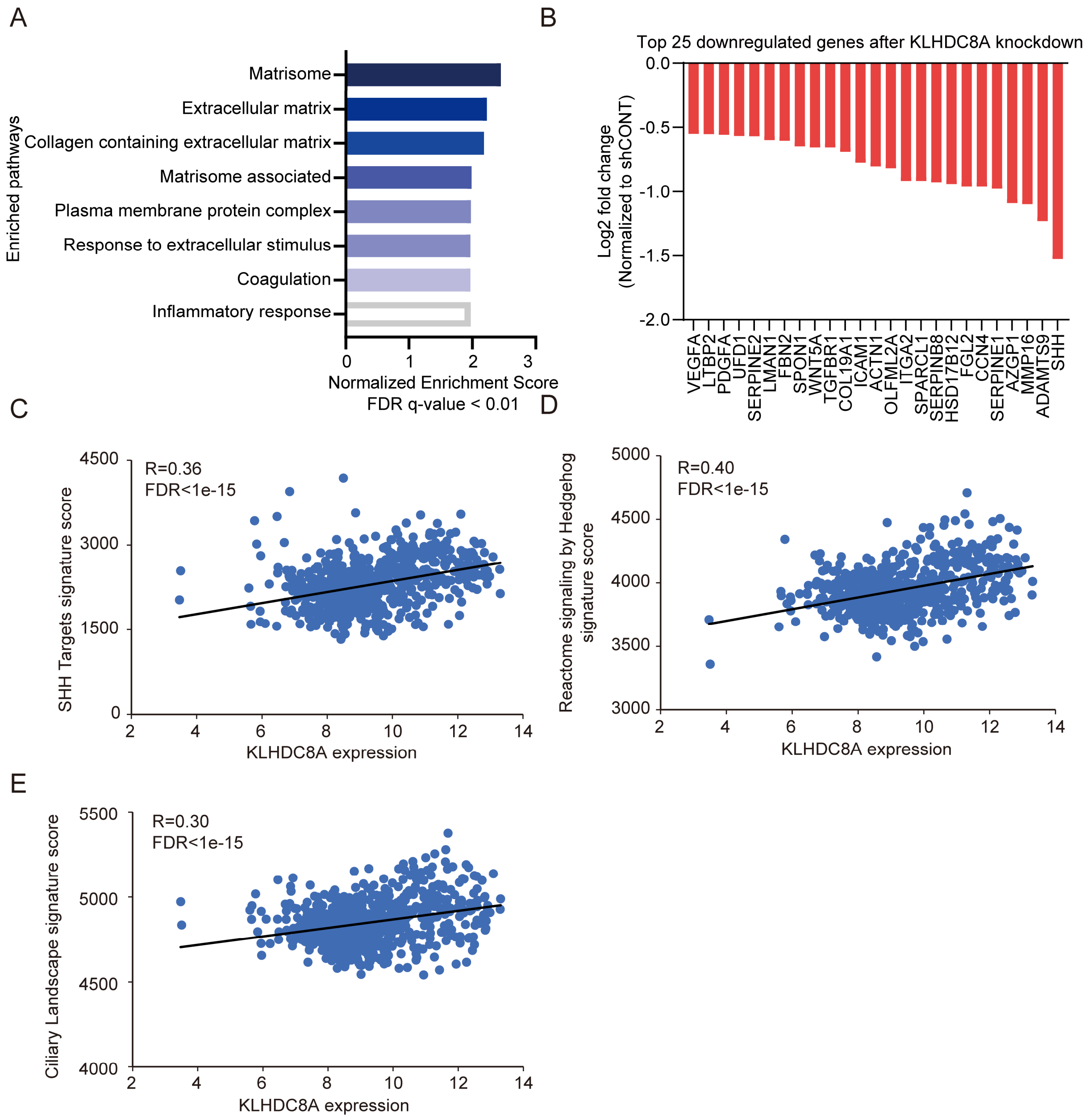

Supplemental Figure 4. KLHDC8A mRNA expression is positively correlated with Shh and Ciliary gene signatures, related to Figures 4 and 5. (**A**) Top 8 downregulated gene sets following KLHDC8A knockdown in GSC23 and GSC3028. Enriched gene signatures are plotted with normalized enrichment score. (**B**) Bar plot showing top 25 downregulated genes from RNA-seq analysis after KLHDC8A knockdown. (**C**) Correlation between KLHDC8A expression and the expression of genes from Shh targets signature. (**D**) Correlation between KLHDC8A expression and the expression of genes from Reactome signaling by Hedgehog signature. (**E**) Correlation between KLHDC8A expression and the expression of genes from Ciliary Landscape signature.

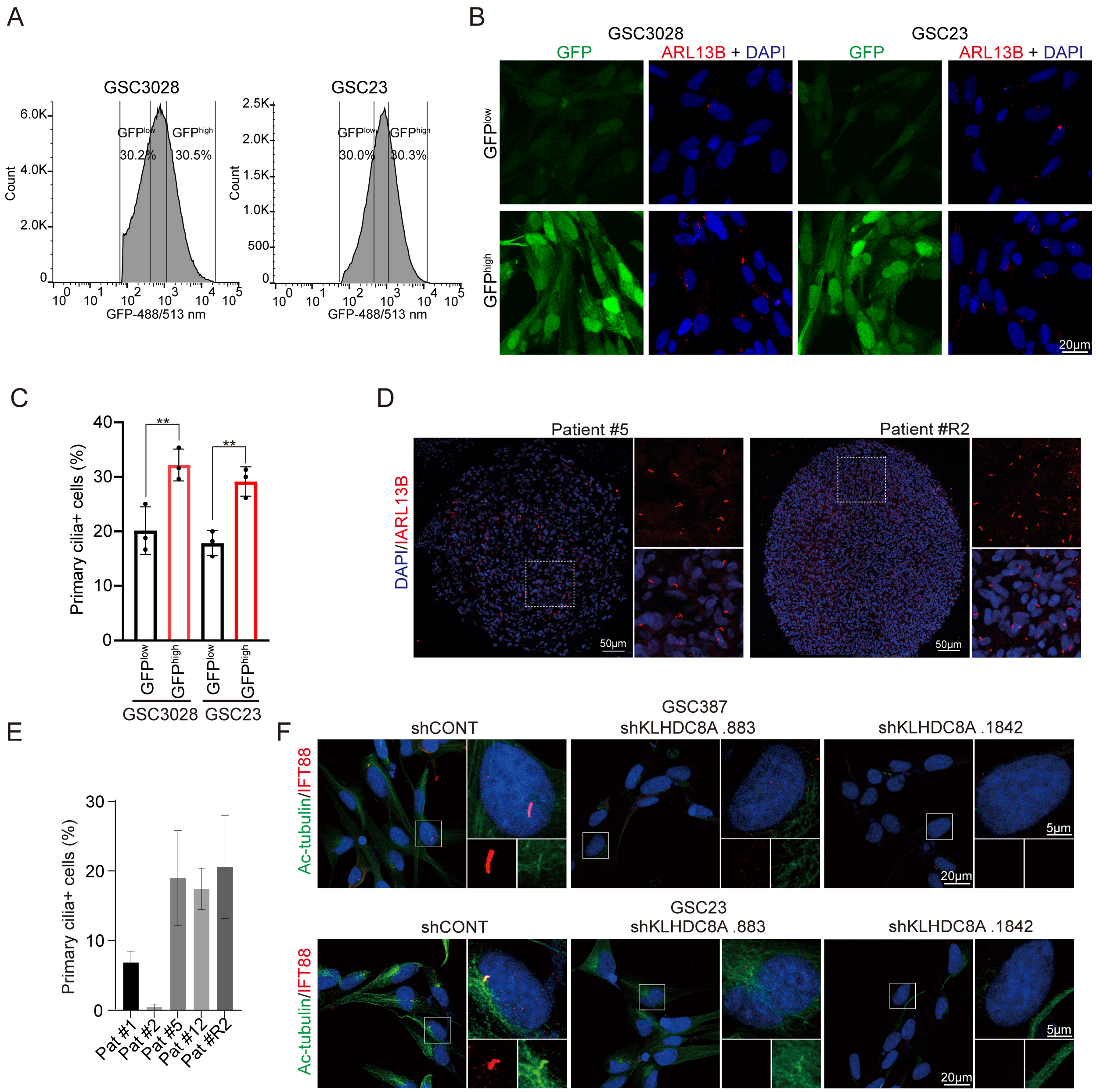

Supplemental Figure 5. Knockdown of KLHDC8A led to loss of primary cilia, related to Figure 7.
(**A**) Fluorescence activated cell sorting (FACS) of GSC3028 and GSC23 transduced with a SOX2 promoter reporter expressing EGFP into GFP^{low} and GFP^{high} subpopulations. (**B**) Immunofluorescence imaging of primary cilia in GFP^{low} and GFP^{high} GSC3028 and GSC23. GFP was showned as green, ARL13B in red, and DAPI in blue. Scales bars represent 5 or 20 µm. (**C**) Quantification of primary cilia positive GFP^{low} and GFP^{high} GSC3028 and GSC23 . At least 50 cells in each GSC line from 3 independent experiments were tested. Quantitative data are shown as mean ± SD (error bars). Statistical analysis was performed using one-way ANOVA with Holm–Sidak multiple test correction. ** $P < 0.01$. (**D**) Representative immunofluorescent labeling of primary cilia in patient-derived tumoroids derived from biopsies of glioblastoma patients. ARL13B (red) is used to label primary cilia, and DAPI is used to label the nucleus. Scale bars = 50 µm. (**E**) Quantification data demonstrated the percentages of primary cilia-positive cells in 5 patient-derived tumoroids. Error bars represent S.E.M. (**F**) Immunofluorescence imaging of primary cilia in GSC387 and GSC23 transduced with shCONT or two non-overlapping shRNAs targeting KLHDC8A. Ac-tubulin was labeled as green, IFT88 as red, and DAPI as blue. Scales bars represent 5 or 20 µm.

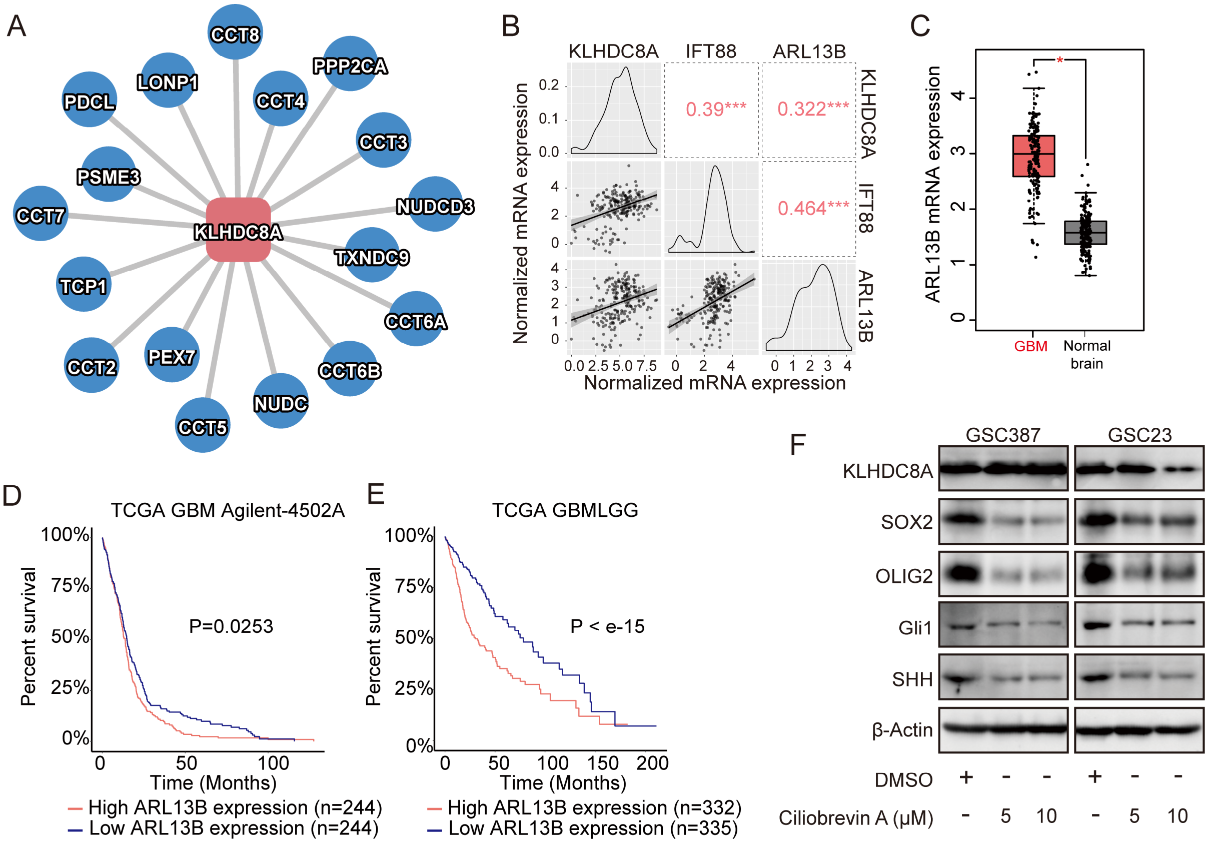

Supplemental Figure 6. ARL13B is upregulated in glioblastoma tissues and informs poor patient prognosis, related to Figure 7. (**A**) Interaction network analysis of KLHDC8A and KLHDC8A binding proteins. The node in red (rectangular) represents KLHDC8A and the nodes in blue (circle) represent KLHDC8A binding partners. (**B**) Correlation of mRNA expression between KLHDC8A, IFT88, and ARL13B in the CGGA glioblastoma dataset. Numbers indicated the R-value of Spearman correlation. *** $P < 0.001$. (**C**) mRNA expression (TPM) of KLHDC8A in normal brain (GTEx dataset, n = 207) and glioblastoma (TCGA dataset, n = 163) from RNA-seq data. Four-way ANOVA was performed to control for sex, age, and ethnicity with Benjamini-Hochberg false discovery rate (FDR) method. (**D**) Kaplan-Meier curve displaying survival of patients in TCGA GBM Agilent-4502A dataset stratified based on median mRNA expression of ARL13B. Statistical analysis was performed using log-rank analysis. $P = 0.0253$. (**E**) Kaplan-Meier curve displaying survival of patients in TCGA GBMLGG glioblastoma dataset stratified based on median mRNA expression of ARL13B. Statistical analysis was performed using log-rank analysis. $P < 1e-15$. (**F**) Immunoblot showing protein levels of KLHDC8A, SOX2, OLIG2, Gli1, and SHH following Ciliobrevin A treatment.

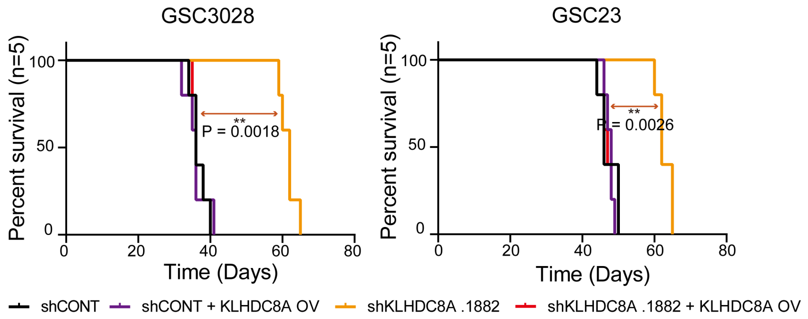

Supplemental Figure 7. Expression of exogenous KLHDC8A in KLHDC8A-depleted GSCs restored in vivo tumor growth of GSCs, related to figure 8.
Kaplan-Meier curve showing survival of NSG immunocompromised mice following implantation with GSC23 or GSC3028 following KLHDC8A knockdown, KLHDC8A overexpression, and exogenous KLHDC8A expression. n=5 per group. Statistical analysis was performed using Mantel-Cox log-rank test. ** $P < 0.01$.

Supplemental Table 1. Molecular subtypes of GSCs used in the manuscript.

Glioblastoma stem cells	Patient Age (Years)	Patient Sex	Tumor Grade	Molecular Subtype
GSC387	76	Female	Glioblastoma (Grade IV)	Classical
GSC3028	63	Female	Recurrent Glioblastoma (Grade IV)	Classical
GSC23	65	Male	Recurrent Glioblastoma (Grade IV)	Classical

Supplemental Table 2: Antibodies used in this study

All the antibodies used in this study are listed

Antigen	Host	Vendor	Catalogue#	Dilution (WB)	Dilution (IF)
KLHDC8A	Rabbit	Novus	NBP1-31181	1:1000	
PARP	Rabbit	Cell signaling	9532	1:1000	
OLIG2	Mouse	Millipore	MABN50	1:1000	
SOX2	Rabbit	AB5604	AB5603	1:1000	
IFT88	Rabbit	Proteintech	13967-1-AP	1:1000	1:200
ARL13B	Rabbit	Proteintech	17711-1-AP	1:1000	1:200
Gli1	Rabbit	Cell signaling	2534T	1:1000	
SHH	Rabbit	Proteintech	20697-1-AP	1:1000	
Acetylated- α -tubulin(Lys40	Mouse	Proteintech	66200-1-lg	1:1000	1:200
α -tubulin	Mouse	Cell signaling	3873S	1:5000	
TCP1	Rabbit	Abcam	Ab225702	1:1000	
β-actin	Rabbit	Proteintech	HRP-60008	1:40000	
GT335	Mouse	Adipogen	AG-20B-0020-C100		1:200
Phospho-Aurora A (Thr288)/Aurora B (Thr232)/Aurora C (Thr198) (D13A11) XP®	Rabbit	Cell signaling	2914	1:500	
Aurora B/AIM1 Antibody	Rabbit	Cell signaling	3094T	1:1000	
GFAP	Rabbit	Proteintech	16825-1-AP	1:1000	

Supplemental Table 3: DNA oligos used in this study

All the DNA oligos used in this study are listed

**Primer oligos for quantifying gene expression**		
**Target**	**Strand**	**Sequence (5'->3')**
KLHDC8A	Forward	ATGGAGGTGCCTAACGTCAAG
	Reverse	CCGTTGTCGTCACATCCCC
SOX2	Forward	GCCGAGTGGAAACTTTTGTCG
	Reverse	GGCAGCGTGTACTTATCCTTCT
OLIG2	Forward	CCAGAGCCCGATGACCTTTTT
	Reverse	CACTGCCTCCTAGCTTGTCC
GFAP	Forward	CTGCGGCTCGATCAACTCA
	Reverse	TCCAGCGACTCAATCTTCCTC
SHH	Forward	CTCGCTGCTGGTATGCTCG
	Reverse	ATCGCTCGGAGTTTCTGGAGA
GLI1	Forward	AGCGTGAGCCTGAATCTGTG
	Reverse	CAGCATGTACTGGGCTTTGAA
CCND1	Forward	GCTGCGAAGTGGAAACCATC
	Reverse	CCTCCTTCTGCACACATTTGAA
CCND2	Forward	ACCTTCCGCAGTGCTCCTA
	Reverse	CCCAGCCAAGAAACGGTCC
CCNE1	Forward	AAGGAGCGGGACACCATGA
	Reverse	ACGGTCACGTTTGCCTTCC
c-MYC	Forward	GGCTCCTGGCAAAAGGTCA
	Reverse	CTGCGTAGTTGTGCTGATGT
CXCR4	Forward	ACTACACCGAGGAAATGGGCT
	Reverse	CCCACAATGCCAGTTAAGAAGA
FOXM1	Forward	CGTCGGCCACTGATTCTCAAA
	Reverse	GGCAGGGGATCTCTTAGGTTC
c-JUN	Forward	TCCAAGTGCCGAAAAAGGAAG
	Reverse	CGAGTTCTGAGCTTTCAAGGT
GAPDH	Forward	GGAGCGAGATCCCTCCAAAAT
	Reverse	GGCTGTTGTCATACTTCTCATGG
**DNA oligos for shRNA**		
**Target**	**TRC number**	
KLHDC8A	TRCN0000138219	
	TRCN0000138761	
ARL13B	TRCN0000381968	
	TRCN0000381442	
SOX2	TRCN0000355694	
	TRCN0000355638	
**DNA oligos for CRISPR Cas9-KRAB gRNA**		
**Target**	**Strand**	**Sequence (5'->3')**
Non-targeting	Forward	CACCGCTCTGCTGCGGAAGGATTCG
	Reverse	AAACCGAATCCTTCCGCAGCAGAGC
	Forward	CACCGCGCGGGGTGGCGATCAATGGAGG

KLHDC8A SE gRNA1	Reverse	AAACCCTCCATTGATCGCCACCCCGCGC
KLHDC8A SE gRNA2	Forward	CACCGGAACGCGGGGTGGCGATCAATGG
	Reverse	AAACCCATTGATCGCCACCCCGCGTTCC
KLHDC8A SE gRNA3	Forward	CACCGGGCGATCAATGGAGGATTACCGG
	Reverse	AAACCCGGTAATCCTCCATTGATCGCCC
KLHDC8A SE gRNA4	Forward	CACCGTTGTTCCAGCCGAAATTAGCAGG
	Reverse	AAACCCTGCTAATTTCGGCTGGAACAAC
KLHDC8A SE gRNA5	Forward	CACCGATTACCGGAAGATGTGCAAATGG
	Reverse	AAACCCATTTGCACATCTTCCGGTAATC

Full unedited blots for Figure 2

Figure 2I:

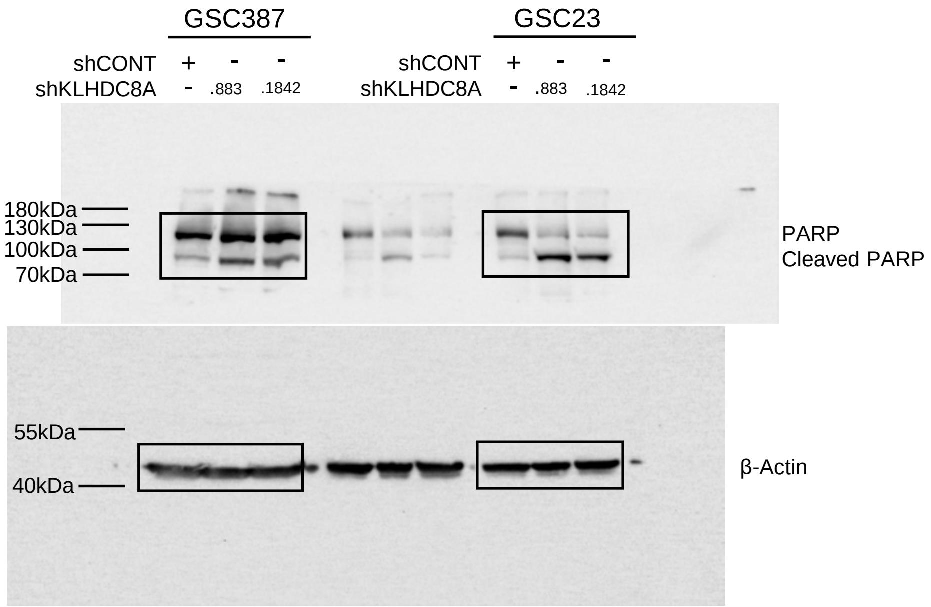

Figure 2J:

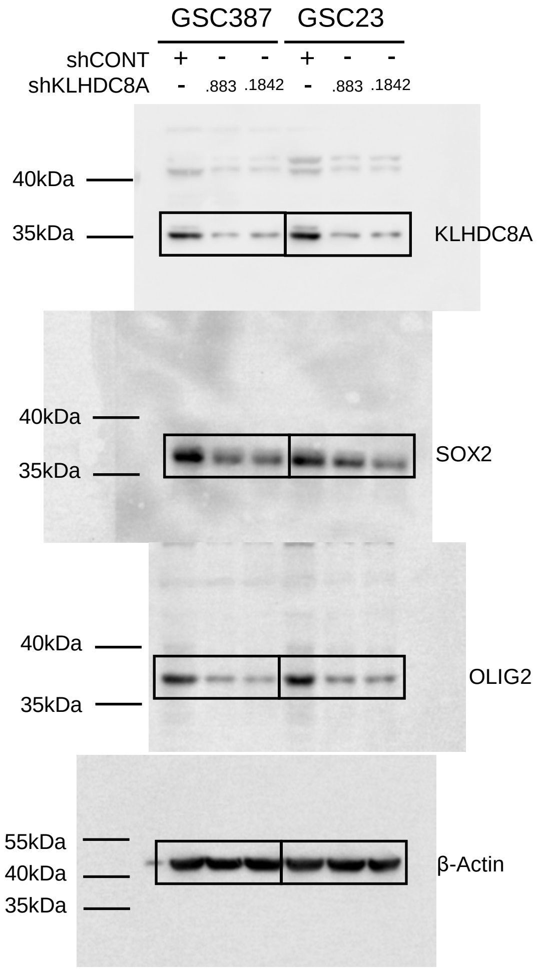

Figure 2K:

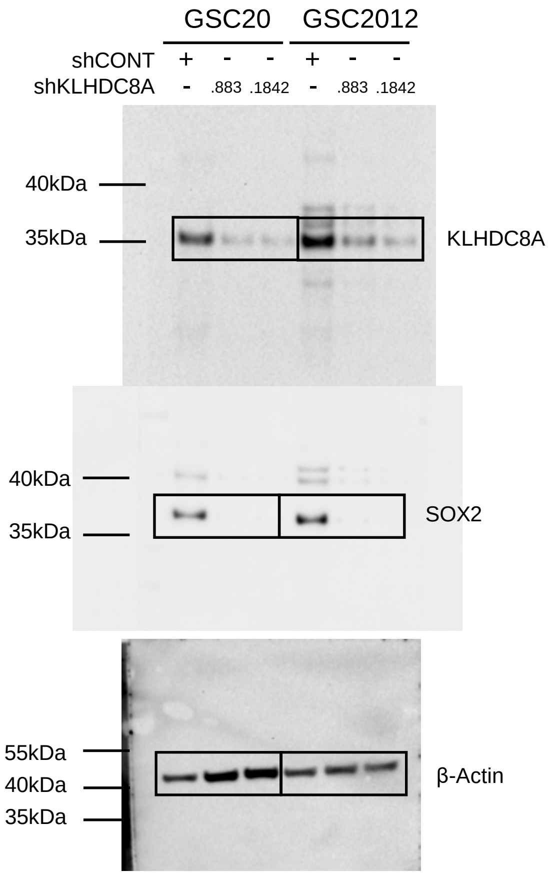

Full unedited blots for Figure 3

Figure 3B:

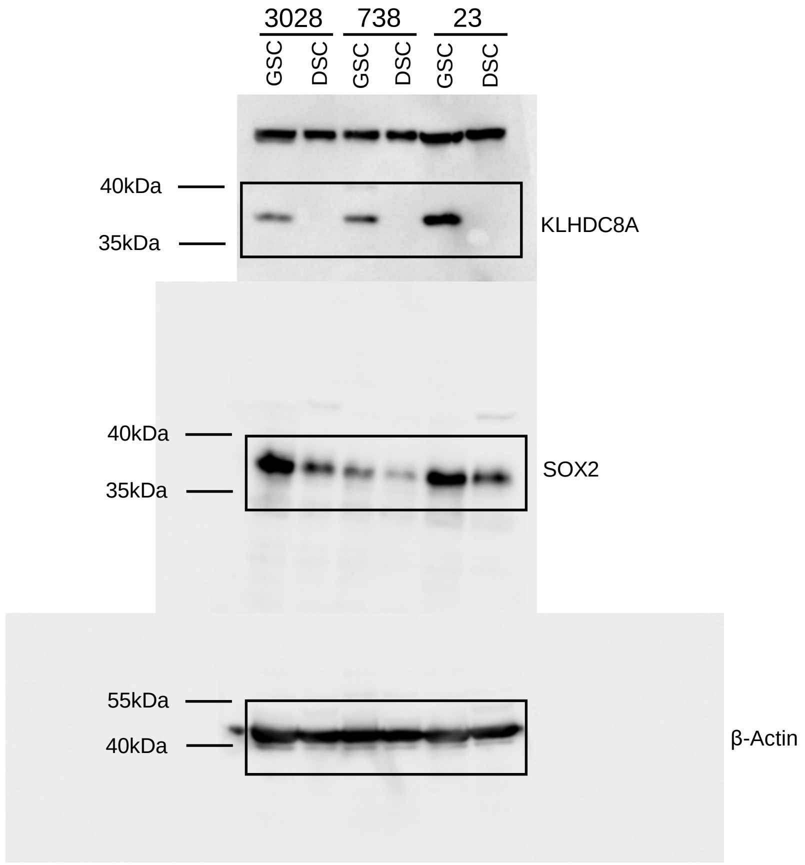

Full unedited blots for Figure 5

Figure 5C:

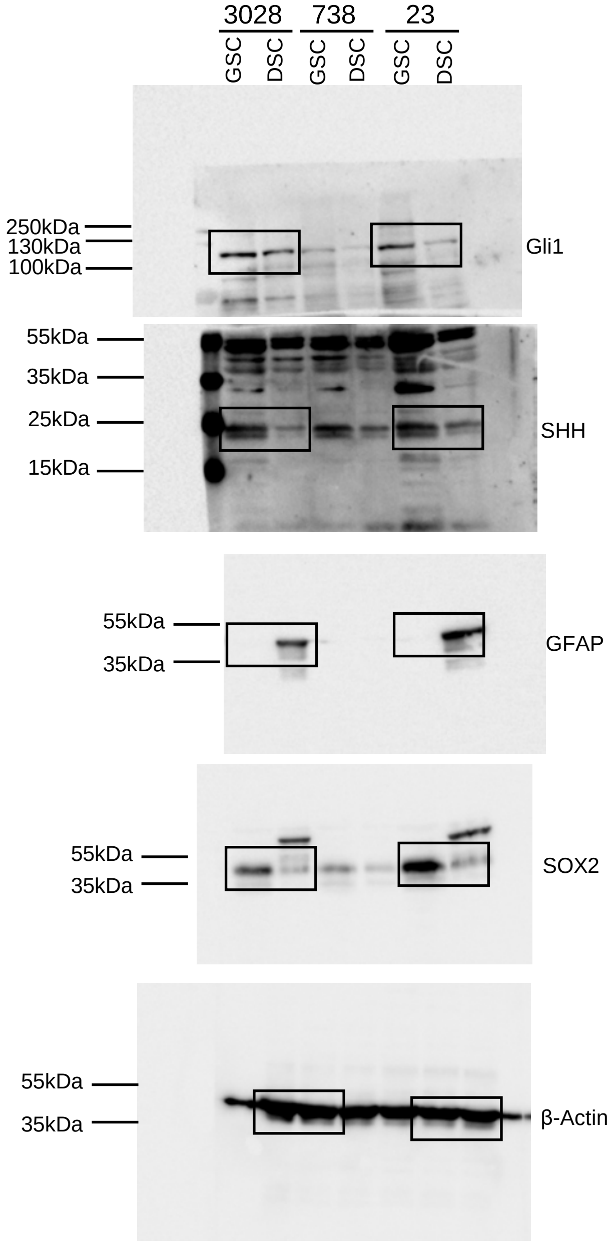

Figure 5F:

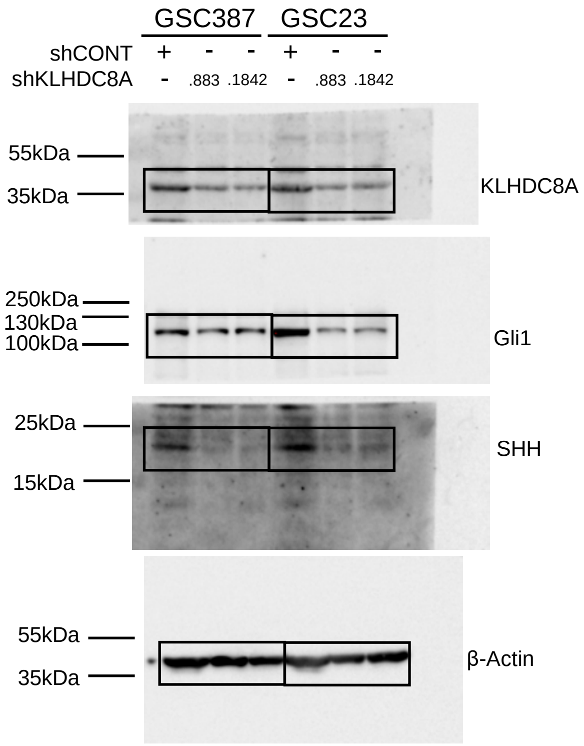

Full unedited blots for Figure 7

Figure 7E:

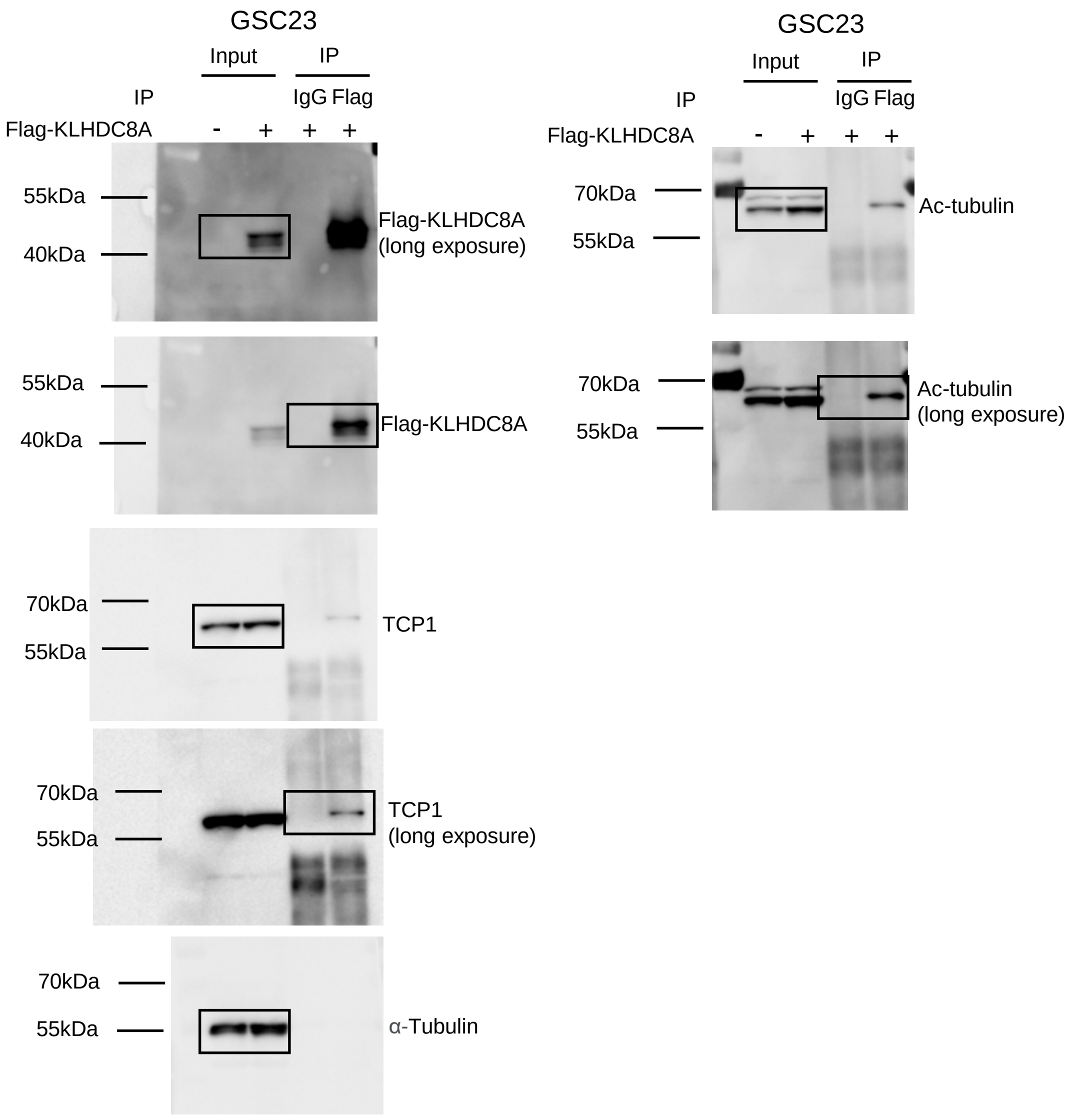

Figure 7F:

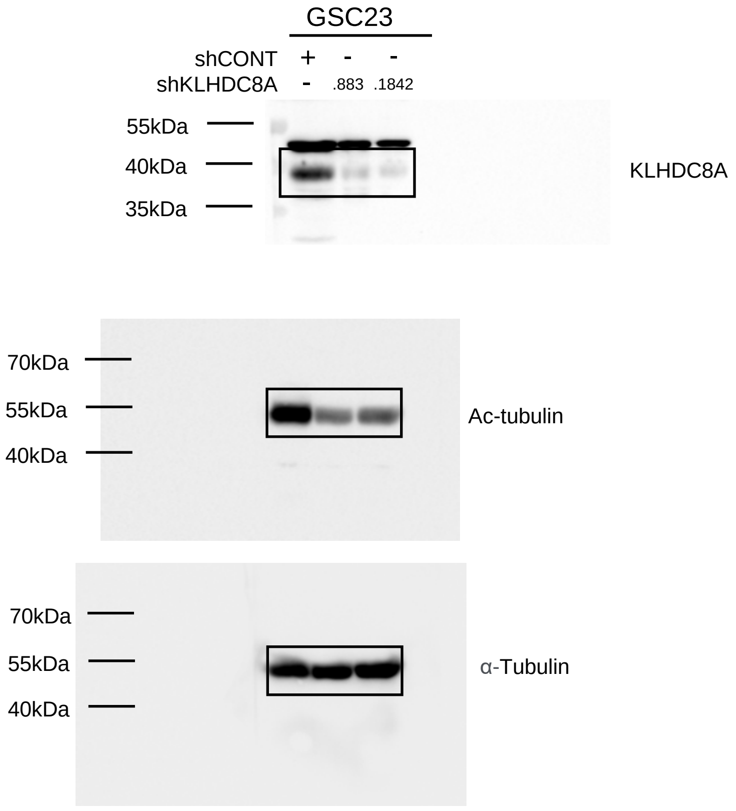

Full unedited blots for Figure 7

Figure 7G:

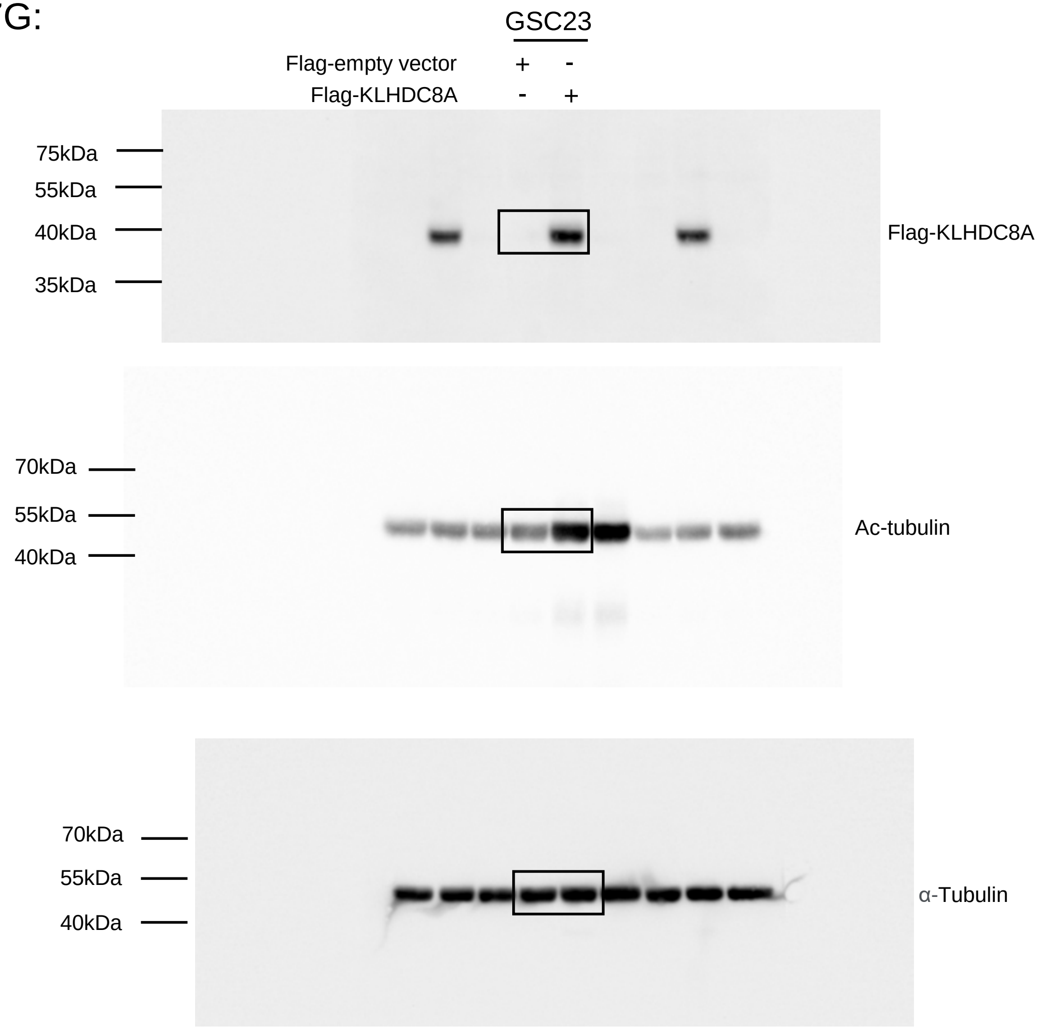

Figure 7H:

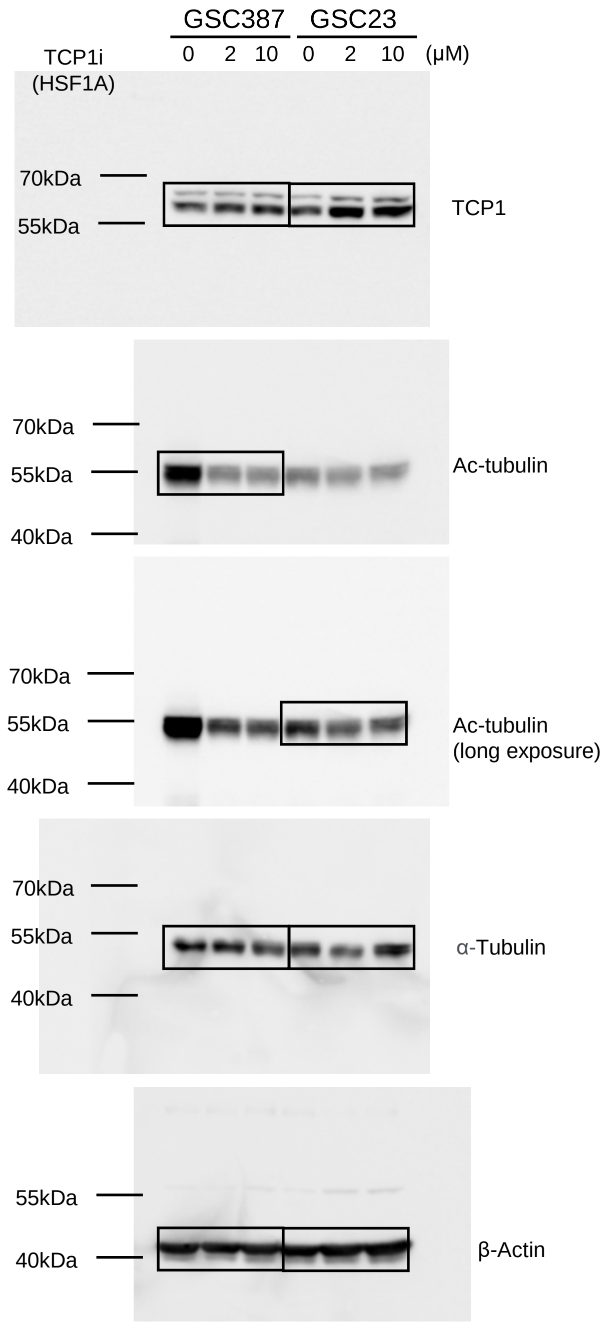

Full unedited blots for Figure 7

Figure 7I:

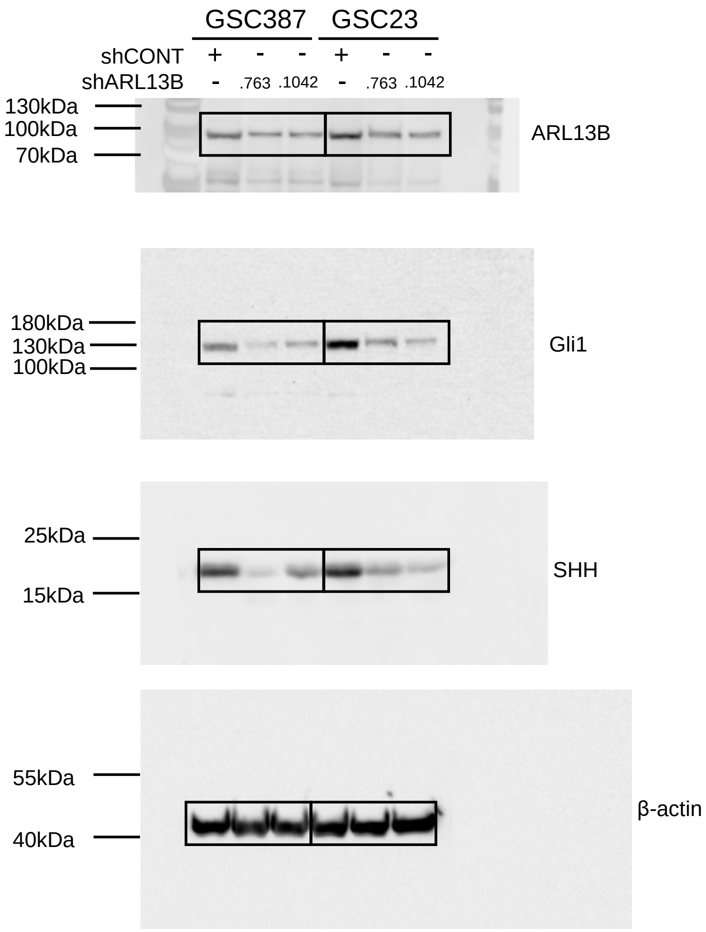

Full unedited blots for Figure 8

Figure 8E:

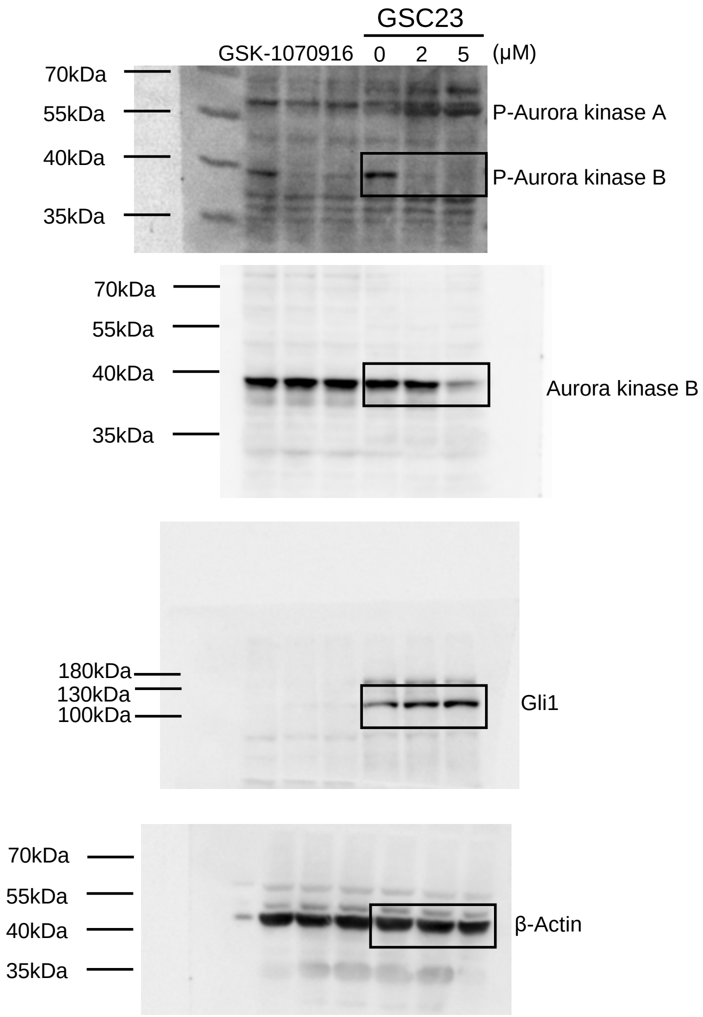

Full unedited blots for Supplemental Figure 2

Supplemental Figure 2B:

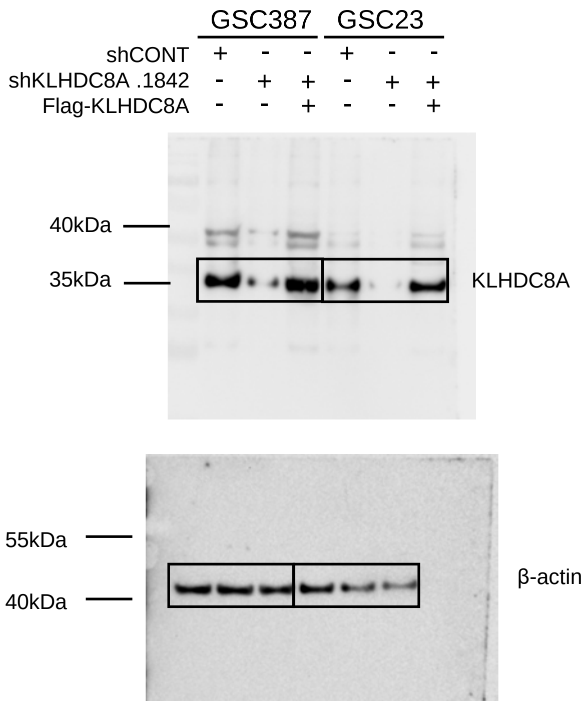

Full unedited blots for Supplemental Figure 6

Supplemental Figure 6F:

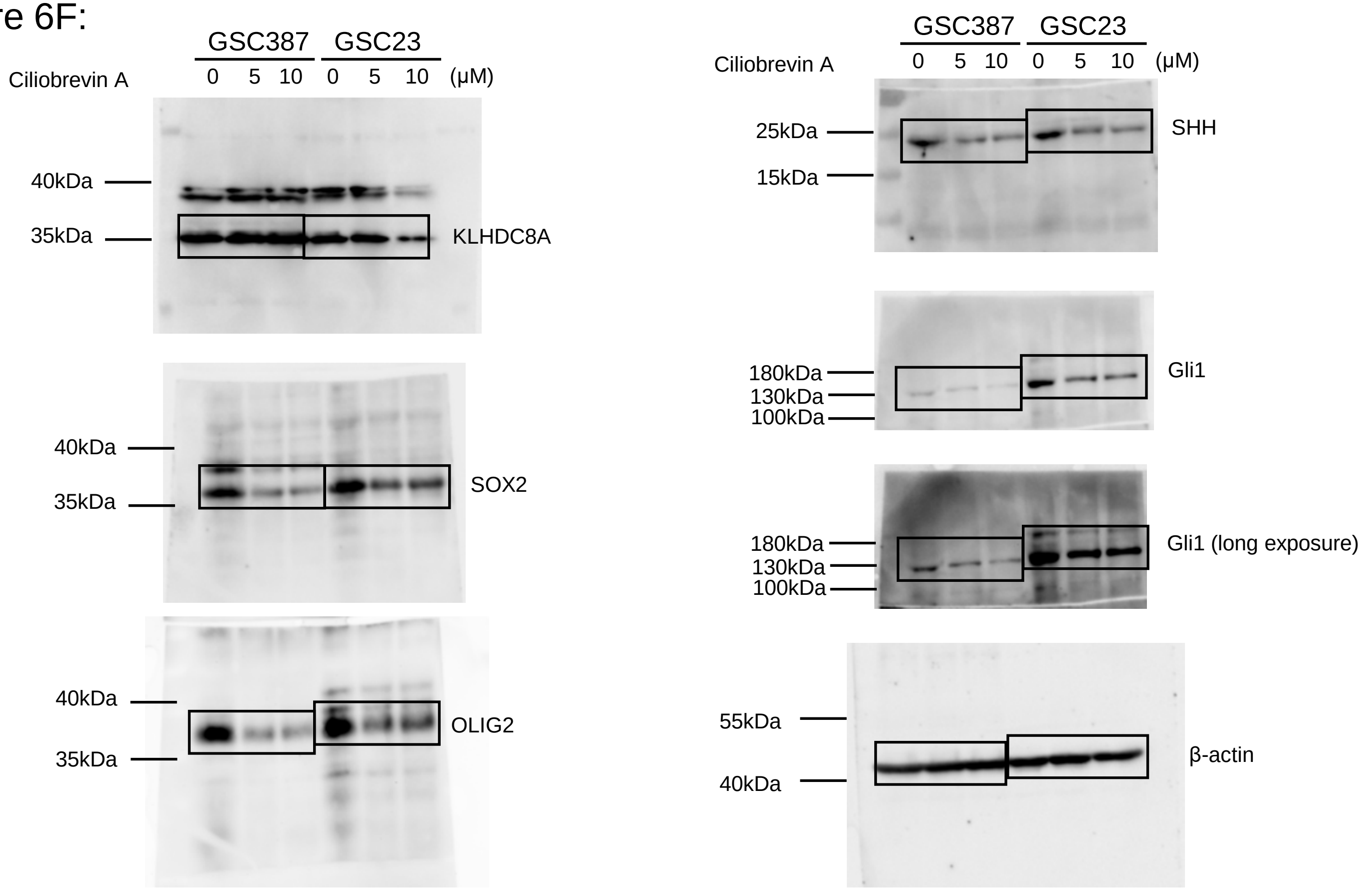

Supplement: Supplemental data [file jci-133-163592-s159.pdf]
